# Treatment With Endothelin-A Receptor Antagonist BQ123 Attenuates Acute Inflammation in Mice Through T-Cell-Dependent Polymorphonuclear Myeloid-Derived Suppressor Cell Activation

**DOI:** 10.3389/fimmu.2021.641874

**Published:** 2021-03-22

**Authors:** Ziyang Chen, Xiaogang Zhang, Shuaijun Lv, Zhe Xing, Mengyu Shi, Xinyao Li, Meiqi Chen, Shaowen Zuo, Yingxu Tao, Gang Xiao, Jingping Liu, Yumei He

**Affiliations:** ^1^Department of Immunology, School of Basic Medical Sciences, Southern Medical University, Guangzhou, China; ^2^Department of Clinical Laboratory, The Third Affiliated Hospital of Southern Medical University, Southern Medical University, Guangzhou, China; ^3^Guangdong Provincial Key Laboratory of Single Cell Technology and Application, Southern Medical University, Guangzhou, China; ^4^Guangdong Provincial Key Laboratory of Proteomics, Southern Medical University, Guangzhou, China

**Keywords:** endothelin-A receptor antagonist, BQ123, acute inflammation, T-cell dependent manner, polymorphonuclear myeloid-derived suppressor cells

## Abstract

The endothelin-A receptor antagonist BQ123 is an effective treatment agent for hypertension and obese cardiomyopathy. However, the role of BQ123 in controlling acute inflammatory diseases and its underlying mechanisms are not well understood. Here, we showed that BQ123 activated polymorphonuclear myeloid-derived suppressor cells (PMN-MDSCs) in mice and that the IL13/STAT6/Arg1 signaling pathway is involved in this process. Importantly, both treatment with BQ123 and the transfer of BQ123-induced PMN-MDSCs (BQ123-MDSCs) were effective in relieving inflammation, including dextran sulfate sodium (DSS)-induced colitis, papain-induced pneumonia, and concanavalin A (ConA)-induced hepatitis, in mice. The treatment effects were mediated by the attenuation of the inflammation associated with the accumulation of PMN-MDSCs in the colon, lung, and liver. However, concurrent injection of Gr1 agonistic antibody with BQ123 induced PMN-MDSC aggravated the observed acute inflammation. Interestingly, no remission of inflammation was observed in Rag2 knockout mice administered BQ123-MDSCs, but co-injection with CD3^+^ T cells significantly relieved acute inflammation. In summary, BQ123-induced PMN-MDSCs attenuated acute inflammation in a T cell-dependent manner, providing a novel potential strategy to prevent the occurrence of acute inflammation.

## Introduction

Endothelin-1 (ET1) and the endothelin-A receptor (ETAR) system play important roles in the pathophysiological progression of cardiovascular diseases (1–3). Endothelin-A receptor antagonists (ETRAs) are critical for the clinical treatment of cardiovascular and vascular diseases such as hypertension, chronic heart failure, pulmonary hypertension, atherosclerosis, and obesity cardiomyopathy (2, 4, 5). Recently, BQ123, a broadly used selective ETRA, has been reported to reduce infarction following focal cerebral ischemia and to relieve pulmonary vascular resistance in congenital heart disease by regulating the ET1/ETAR system (6, 7). Evidence also suggests that the ET1/ETAR system is involved in the pathogenesis of severe inflammation and septic shock (8). The ET1/ETAR system blocking with BQ123 may have clinical applications in the treatment of septic shock (9). It has also been reported that BQ123 can reduce the expression of proinflammatory cytokines, including reactive oxygen species (ROS), tumor necrosis factor (TNF)-α, interleukin (IL)-1, and IL-6, by regulating the ET1/ETAR system (10–12). BQ123 may also play a role in alleviating chronic airway inflammatory obstructive asthma by reducing fibrocyte differentiation and progression (13). Another ETAR antagonist, atrasentan, has been shown to control colitis through its effects on the ET1-ETAR system (14). However, the role of BQ123 in the direct regulation of acute immune-mediated inflammatory diseases and the underlying mechanisms remain largely unclear.

Myeloid-derived suppressor cells (MDSCs) are a heterogeneous population of pathologically activated immature myeloid cells and an abnormal accumulation of myeloid progenitor cells. There are two subsets of MDSCs: polymorphonuclear MDSCs (PMN-MDSCs), which have a CD11b^+^Ly6G^+^Ly6C^Low/−^phenotype; and monocytic MDSCs (M-MDSCs), which have a CD11b^+^Ly6G^−^Ly6C^High^ phenotype (15, 16). Compared with monocytes and neutrophils, these subgroups share the same origin, morphology, and phenotypic characteristics. However, they exhibit relatively immature phenotypes and morphology, and have powerful immunosuppressive functions. These are targeted toward various immune cells, in particular T-cells, and some specific target genes such as intracellular ROS, arginase (Arg1), prostaglandin E2 (PGE2), S100A8/9, and a series of inflammatory cytokines (17–20). Initial research focused on the role of MDSCs in the negative regulation of the immune response, in the context of pathological conditions such as cancer, autoimmunity, inflammatory conditions, trauma, and infection (21–23). The discovery of MDSCs represents a new avenue for the clinical treatment of tumors (19–21). Furthermore, recent research has demonstrated that PMN-MDSCs occur in pregnancy and newborns (24–27). In addition, many studies have shown that MDSCs can be used to relieve rheumatoid arthritis, asthma, and multiple sclerosis (28–31). However, the regulatory activities of MDSCs in the context of treating inflammatory diseases have not been fully investigated.

Inflammation is a beneficial automatic defense response that protects the body from potential harm caused by infection, injury, or autoimmune damage (32). However, inflammation can also have negative consequences; over-reactive leukocyte infiltration and severe inflammatory cytokine reactions can result in whole-body tissue damage (33–36). In many systems, without effective intervention, persistent inflammation promotes inflammation-driven carcinogenesis, which is the most common cause of death worldwide (37–39). Existing research sheds light on the treatment of inflammation, including the roles of nonsteroidal anti-inflammatory drugs (NSAIDs), glucocorticoids, IL-1β neutralization, and cellular immunotherapy. However, many anti-inflammatory drugs have severe side effects (40–42). Therefore, the use of cellular immunotherapy for the treatment of inflammation is promising. It has been reported that freshly cultured allogeneic bone-marrow derived mesenchymal stem cells are safe to administer to patients with septic shock (43). Several studies have also shown that MDSCs can be used to control necrotizing enterocolitis (NEC) (24, 44). However, the effect of BQ123 on immune mechanisms in the context of treating inflammatory diseases requires further study.

This study revealed that treatment with BQ123 promoted PMN-MDSC activation in mice, and that the IL13/STAT6/Arg1 signaling pathway was involved in this process. Moreover, treatment with BQ123 and the transfer of BQ123-induced PMN-MDSCs (BQ123-MDSCs) were effectively alleviated inflammation, including acute colitis, acute pneumonia, and acute hepatitis. However, injection of Gr1 agonistic antibody aggravated the observed acute inflammation. Moreover, BQ123-induced PMN-MDSCs attenuated acute inflammation in a T cell-dependent manner. Collectively, BQ123 and BQ123-MDSCs may serve as a potential therapeutic agent for acute immune-mediated inflammation.

## Materials and Methods

### Mice

BALB/c and C57BL/6J mice were purchased from the Laboratory Animal Center of the Southern Medical University. Rag2 KO mice (B6.129-Rag2tm1) were purchased from the Cavens Biogel (Suzhou) Model Animal Research Co. Ltd. All mice were housed in pathogen-free facilities under the following conditions: temperature 23 ± 2°C, humidity, 55 ± 5%, 12 h light/dark cycle. All mice had free access to standard rodent pellet food and tap water according to the Southern Medical University guidelines. All experimental procedures in this study were approved by the Institutional Animal Care and Use Committee of the Southern Medical University Experimental Animal Ethics Committee (STAMP) (Approval number: L2019130).

### Reagents and Antibodies

The reagents and antibodies used in this study are listed in **Supplementary Tables 1** and **2** respectively.

### Cell Isolation

#### Isolation of Mouse Bone Marrow (BM) Cells

Bone marrow cells were obtained by flushing femurs and tibias with a 20mL syringe containing RPMI-1640 medium followed by lysing of red blood cells with Ammonium-Chloride-Potassium (ACK) buffer.

#### Isolation of Lamina Propria Mononuclear Cells (LPMCs)

Following the removal of feces, the colon was cut into 1 cm pieces using scissors, and incubated in 5 mL of pre-digestion HBSS buffer (1 mM DTT, 5 mM EDTA, and 20 mM HEPES) for 20 min at 37°C under slow rotation in a shaker, to remove intestinal epithelial cells. Subsequently, the colon was digested in 0.5 mg/mL collagenase I and 5 U/mL DNase for 60 min to isolate the LPMCs. Leukocytes from the lamina propria were enriched using a 40%/80% Percoll gradient (GE Healthcare).

#### Isolation of Mononuclear Cells From Lung Tissue

To isolate cells from the lung tissue, we flushed the lungs with 1 mL of cold PB twice, using a thin tube inserted into a cut made in the trachea, using methods previously described by Monticelli et al. (45). The lungs were then perfused with 20 mL cold phosphate-buffered saline (PBS) through the right ventricle of the heart before removal. Lung lobes were cut into small pieces using scissors and were digested with 0.5 mg/mL collagenase type I (Invitrogen) in RPMI-1640 medium with 10% FBS (Biological Industries) and 1% penicillin-streptomycin (Gibco) for 1 h at 37°C, and were subjected to continuous agitation in a shaker. Leukocytes were obtained using a 40/80% Percoll gradient (GE Healthcare).

#### Isolation of Mononuclear Cells From Liver

Leukocyte cells were obtained from the liver using mechanical disruption and 70 µm cell strainers. Hepatic mononuclear cells were isolated using a 30%/70% Percoll gradient (GE Healthcare).

For all samples, the middle layer was gently taken out using a Pasteur pipette and washed with PBS twice after density gradient centrifugation at 400 ×g and 25°C for 25 min. Red blood cells were lysed in ACK buffer, and cell suspensions were filtered through 70 μm cell strainers before subsequent analysis.

### Flow Cytometric Analysis

Cells were first stained with fluorescein-conjugated Ghost Dye Violet 780 to exclude dead cells. Then, surface markers were then stained for 30 min at 4°C. For staining of transcription factors, cells were fixed and permeabilized according to the manufacturer’s instructions, after staining with surface antibodies. To measure intracellular cytokine expression, cells were isolated and stimulated in complete RPMI-640 medium + 10% FBS with 50 ng/mL PMA, 1 µg/mL ionomycin, and 1 µg/mL brefeldin A for 4 h. Cells were subsequently surface-stained, fixed, and permeabilized using a CytoFix/Perm solution, and stained with the indicated cytokines. All the cells were protected from light during the dyeing process. An LSR Fortessa flow cytometer (BD Bioscience) was used for all flow cytometry data acquisition, and data were analyzed using FlowJo V10.0.7. Flow cytometry was supported by the Department of Immunology at the School of Basic Medical Sciences, Southern Medical University. The gating strategy for mouse MDSCs was CD11b^+^Ly6G^+^Ly6C^Low/-^ for PMN-MDSCs and CD11b^+^ Ly6G^–^Ly6C^High^ for M-MDSCs. Examples of MDSC gating strategies are provided in **Supplementary Figure 1**.

### Cell Sorting

For Ly6G^+^ cells isolation, the cells were labeled with anti-mouse biotin Ly6G antibodies (catalog no. 127604, BioLegend). For CD3^+^ cells isolation, the cells were labeled with anti-mouse biotin CD3 antibodies (catalog no. 100304, eBioscience). The cell–antibody complex suspension was mixed with streptavidin microbeads (catalog no. 130-048-1012, Miltenyi) at 4°C for 30 min. All cells were sorted to a purity of ≥ 95%. Purified immature innate lymphoid cells (iILC2) from BM, cells were initially depleted of T, B, myeloid and erythroid lineages by labeling with biotin-conjugated anti-CD3, anti- B220, anti-CD11b, anti-Ly6G, anti-CD11c, anti-NK1.1, anti-CD4, anti-CD5, anti-CD8a, anti-TCRβ chain, anti- TCR γ/δ, and anti-erythroid marker (TER-119), followed by streptavidin-paramagnetic particles (BD Biosciences) according to the manufacturer’s instructions. The remaining cells were stained with the specific fluorochrome-conjugated antibodies: Lin^-^CD45^+^ Sca1^+^CD25^+^CD127^+^and sorted with Aria III (BD Biosciences).

### T-Cell Proliferation Assay

To evaluate PMN-MDSC suppressive activity, neutrophils and PMN-MDSCs were extracted from the spleen of mice treated with PBS or BQ123, and co-cultured with CD3^+^ T-cells isolated from the spleens of BALB/c mice, labeled with CFSE at 37°C for 15 min. Cells were plated in 96-well plates in RPMI-1640 with 10% FBS at different ratios (T/MDSC 1:0, 2:1, 4:1, and 8:1) and stimulated with concanavalin A (ConA) (5 μg/mL). Unstimulated T-cells were used as a negative control. To block the molecular effector of MDSCs, either the arginase inhibitor Nω-Hydroxy-nor-L-arginine monoacetate (NOHA, 100 μM), the EP2 inhibitor AH6809 (5 μM), the EP4 inhibitor L-161982 (5 μM), or the ROS inhibitor N-acetylcysteine (NAC, 1 mM) were added to T/MDSC 2:1 of each group separately. After 72 h, cells were stained with anti–CD4-PB and anti–CD8a-PE, and T-cell proliferation was assessed based on the intensity of CFSE fluorescence using an LSRFortessa flow cytometer. Examples of gating for T-cell proliferation are provided in **Supplementary Figure 1**.

### PMN-MDSC Induction *In Vivo*

Four-week-old C57BL/6 mice were injected intraperitoneally with BQ123 (5 mg/kg/day in a volume of 200ul 1‰ DMSO/PBS), or ET1 (5 mg/kg/day in a volume of 200ul 1‰ DMSO/PBS) for eight consecutive days. Control mice received the vehicle (1‰ DMSO/PBS) *via* the same route. The mice were euthanized 24 h after receiving the last dose. For the STAT6 inhibitor experiment, the mice were divided into four groups. One group received a PBS injection, the second group received a BQ123 injection, the third group received a combination of BQ123 and AS1517499, and the fourth group received only an AS1517499 injection. AS1517499 was injected intraperitoneally at a dose of 10 mg/kg (dissolved in 1‰ DMSO/PBS) for eight consecutive days, and subsequent experiments were performed on day 9.

### Co-Culture BQ123 With iILC2s

The iILC2s sorted from borrow marrow were cultured (about 1×10^5^ cells in 200 ul RPMI-1640 medium containing 10% fetal bovine serum) in 96-well plates in the presence of IL-2 (20 ng/ml) and IL-7 (20 ng/ml), IL-33 (100 ng/ml) with or without BQ123 (100uM, dissolved in 1‰ DMSO/PBS). Media were half changed on day 3 and the amounts of cytokines of ILC2 in cells (IL-5^+^ IL-13^+^) were analyzed by flow cytometry on day 6. The levels of IL-5 and IL-13 in culture supernatants were also measured by enzyme-linked immunosorbent assay (ELISA).

### Enzyme-Linked Immunosorbent Assay

Cell lysates of PMN-MDSCs and control cells were collected to evaluate PGE2 concentrations (catalog no. E-EL-0034c, Elabscience) and the protein levels of S100A9 (catalog no. DY2065, R&D, USA). The levels of IL-5 and IL-13 in bronchoalveolar lavage fluid (BALF) of mice with acute lung inflammation or culture supernatants from BQ123 co-cultured with iILC2, and those of aspartate aminotransferase (AST) and alanine aminotransferase (ALT) in the serum of mice with acute hepatitis were measured (catalog no. C010-2-1/C009-2-1, njjcbio, China). Thermo Scientific Multiskan FC systems were used to detect levels of PGE2, S100A9, IL-5, IL-13, AST, and ALT; all procedures were performed according to the manufacturer’s instructions.

### ROS Production Assay

Intracellular ROS production was measured by fluorescence microscopy, using 2’,7- dichlorodihydrofluorescein diacetate (DCFHDA) (catalog no. D399, Invitrogen) at a dilution of 1:1,000, according to the manufacturer’s instructions. The cells were kept at 37°C and were not exposed to light. The cells were then stained with antibodies (**Supplementary Table 2**).

### Arginase Activity Assay

The arginase reaction was performed according to the manufacturer’s instructions. Briefly, approximately 1×10^6^ PMN-MDSCs and neutrophils were collected in a 1.5 ml centrifuge tube, to which 100 μL radioimmunoprecipitation assay lysis buffer (pH 7.4) (Beyotime, China) was added to obtain the cell lysate. Cell lysates were incubated at 37°C for 2 h after the addition of L-arginine and MnCl2. Urea (1 mM) was used as the standard sample, and water was used as the blank. After incubation was complete, the absorbance was measured at 450 nm using a spectrophotometer, and the arginase activity was calculated using the formula given in the manufacturer’s instructions.

### Quantitative Real-Time Polymerase Chain Reaction (qRT-PCR)

Total RNA was extracted from cells using TRIzol reagent (catalog no. 15596, Invitrogen). Reverse transcription PCR (RT-PCR) was performed using a ProFlex PCR system (ThermoFisher Scientific) with a StarScript II First-strand cDNA synthesis kit (catalog no. A212-05, GenStar). Real-time quantitative PCR was performed using a QuantStudio 6 Flex system (Thermo Fisher Scientific) and a RealStar Green Power Mixture kit (catalog no. A314-10, GenStar). The mRNA levels of specific genes were determined using the relative standard curve method and used β-Actin for normalization, and the lowest expression level sample in control group was artificially set to 1. qPCR analyses were performed in triplicate, and experiments were repeated at least twice. The primer sequences used are listed in **Supplementary Table 3**.

### Western Blotting

The experimental protocol for western blotting was described previously by He et al. (46). All protein sample was sorted from spleen of mice treated with PBS or BQ123. Cells were lysed with radioimmunoprecipitation assay lysis buffer (Beyotime, China). Protein concentrations were measured using a bicinchoninic acid protein assay kit. The target proteins were separated using SDS-PAGE and transferred onto polyvinylidene fluoride membranes (Millipore, Billerica, MA,USA). After blocking for 1 h, the membranes were incubated with the corresponding primary antibodies at 4°C for 16 h, followed by incubation with HRP-conjugated secondary antibodies at room temperature for 1 h. Protein bands were visualized using a western blotting detection kit (catalog no. WBKLS0500, MILLIPORE), the chemiluminescent signal was detected using ChemiDocTM XRS^+^, and the band intensity was assessed using Image LabTM Software (Bio-Rad). Tubulin and total STAT6 were used as loading controls. The antibodies used in this study are listed in **Supplemental Table 2**.

### DSS-Induced Colitis Model

Murine acute colitis was induced as previously described by Wirtz et al. (47), with the slightly modifications. Briefly, six-week-old C57BL/6 male mice was induced by giving 3% DSS (MW36 000–50 000; MP Biomedicals) dissolved in drinking water for seven consecutive days, and were euthanized by cervical dislocation under isoflurane anesthesia on day 8. To evaluate the severity of colitis, animals were monitored daily for loss of body weight, stool consistency and hematochezia. Individuals were also given a clinical score for the disease activity index (DAI), which ranged from 0 to 4. The entire colon was removed, gently flushed with saline and blotted on filter paper, cleaned of fat and mesentery, and longitudinally opened so as to exhaustively eliminate faucal residues. Each specimen was weighed and its length was measured under a constant load. A fraction of colon tissue was used for histological examination and scored, and remaining colon tissue were measured the expression level of inflammation factors, as well as the population of MDSC and Th subsets in lamina propria. Bacterial loading in the colon was tested as described previously (48–50). DNA samples were amplified using both 16S rRNA universal primers and 16S rRNA gene group-specific primers (UniF340 and UniR514). The primer sequences are presented in **Supplementary Table 3**.

### Papain-Induced Acute Pneumonia Model

For induction of papain-induced pneumonia mouse model (45), mice were anesthetized, followed by intranasal administration with papain (20 µg papain in 40 µL PBS, daily) for five consecutive days. Control mice were treated with an equal volume of PBS only. Twenty-four hours after the final treatment; the mice were euthanized by cervical dislocation under isoflurane anesthesia. Then BAL fluid was collected for analysis the frequency of eosinophils (EOS) and level of IL-5/IL-13. The left lobe of lung was fixed in 4% buffered formalin for histological examination, and remaining lung tissue were measured the expression level of IL5 and IL13.

### ConA-Induced Murine Hepatitis Model

Murine hepatitis was induced using the methodology described by Arshad et al. (51), with slightly modifications. Briefly, mice were administered a single intravenous tail vein injection (i.v.) of ConA (Sigma-Aldrich) at a dose of 13 mg/kg body weight on day 0 and euthanized after 24 h, and the control mice were injected with PBS. Serum was collected from the blood of the eyeball, and the serum levels of AST and ALT in serum were measured by ELISA according to the manufacturer’s instructions (Nanjing Jiancheng Bioengineering Institute, Nanjing, China). And the liver lobule were fixed in 4% buffered formalin for histological examination, and remaining liver tissue were measured the expression level of inflammation factors, as well as the population the level of MDSC and Th1 cells.

### Adoptive Transfer of BQ123-Induced PMN-MDSC

Adoptive transfer of PMN-MDSCs was carried out as described previously by He et al. (24). PMN-MDSCs and neutrophils were purified from the donor mice, which were intraperitoneally administered with BQ123 or PBS for eight consecutive days. Approximately 2×10^6^ PMN-MDSCs or neutrophils were intravenously injected into recipient mice on different days. For the DSS-induced colitis model transfer, 2 × 10^6^ enriched BQ123-induced PMN-MDSCs were injected into recipient mice on days 2 and 5. For the papain-induced lung inflammation mouse model, 2 × 10^6^ enriched BQ123-induced PMN-MDSCs were injected into recipient mice on days 0 and 3; and for the acute hepatitis model, 2 × 10^6^ BQ123-induced PMN-MDSCs were intravenously injected into recipient mice 1 h before receiving the ConA injection on day 0. PBS-treated neutrophils were used as the control. For T cell transfer in Rag2 KO, about 4×10^6^ CD3^+^T cells from donor mice were injected into Rag2 KO recipient mice during inducing different inflammation models in the same cycle. Donor CD3^+^T cells were isolated from the spleen of C57BL/6 WT mice.

### BQ123-Induced PMN-MDSC Depletion

In the BQ123-induced PMN-MDSC depletion experiment, 10 µg/g of anti-Gr1 (BioXcell, West Lebanon, New Hampshire, and USA) was administered intraperitoneally to WT mice. C57BL/6 mice were administered BQ123 or PBS *via* the same route as mentioned above before model induction. For the colitis mouse model, Gr1ab was injected into recipient mice on days 2 and 5 of the model. For the lung inflammation mouse model, Gr1ab was injected into recipient mice on days 0 and 3, for the acute hepatitis model, Gr1ab was injected into recipient mice 1 h before receiving the ConA injection on day 0. The rat IgG isotype was used as a control.

### Statistical Analysis

All experimental data were analyzed using GraphPad Prism version 8.0a (GraphPad Software Inc., San Diego, CA, USA). For most experiments, differences between the two groups were assessed using two-tailed unpaired Student’s t-tests and one-way analysis of variance. Non-parametric Mann-Whitney tests were used to compare the differences between more than two groups when the variances were significantly different. * indicates p-values, and ns denotes non-significance. If a p-value was <0.05, the difference was considered statistically significant. *P-value < 0.05; **P-value < 0.01; ***P-value < 0.001; ****P-value < 0.0005.

## Results

### BQ123 Promoted PMN-MDSC Activation in Mice

**Figure 1:**
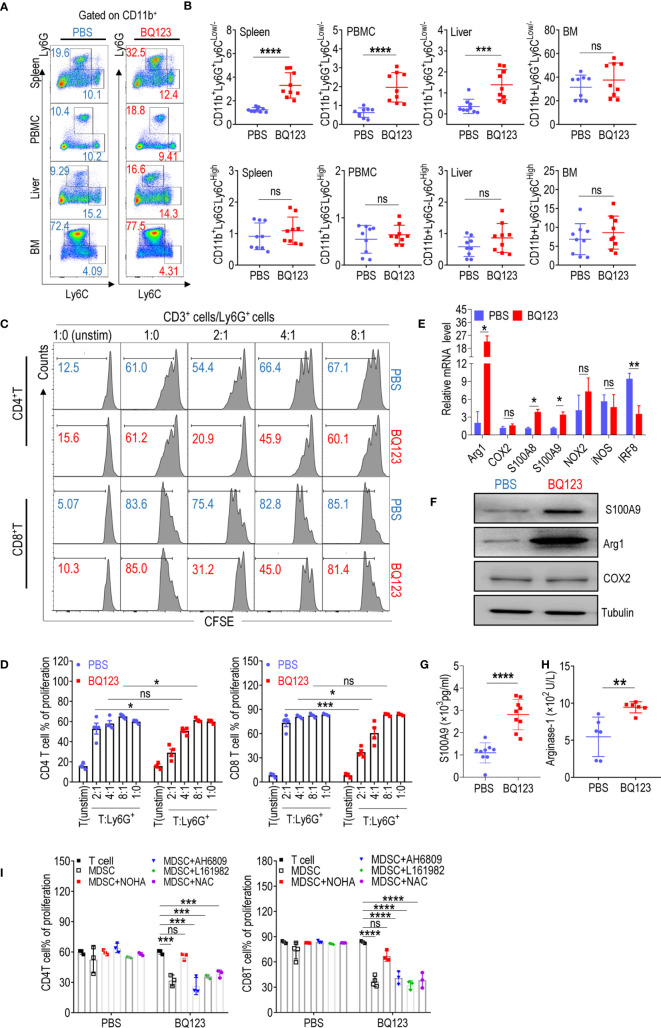
BQ123 promoted PMN-MDSCs activation in mice. **(A, B)** C57BL/6 mice (four-weeks of age) were injected intraperitoneally with BQ123. Control mice received PBS *via* the same route. Mice were euthanized after 8 days and tissues were obtained. The proportions of MDSC subsets (CD11b^+^Ly6G^+^Ly6C^Low/-^ for PMN-MDSCs and CD11b^+^ Ly6G^–^Ly6C^High^ for M-MDSCs) in the spleen, peripheral blood mononuclear cells (PBMCs), liver, and bone marrow (BM) were measured using flow cytometry analysis. A typical example of flow cytometry **(A)** and statistical results from multiple experiments **(B)** are shown (n=9). **(C, D)** The suppressive activity of BQ123 induced CD11b^+^Ly6G^+^Ly6C^Low/-^ cells were determined. CD11b^+^Ly6G^+^Ly6C^Low/-^ cells and neutrophils were sorted from the spleen of mice treated with BQ123 or PBS, and co-cultured with CD3-T cells isolated from the spleens of BALB/c mice labeled with CFSE at 37°C for 15 min. Cells were plated in a 96-well plate in RMPI-1640 with 10% FBS at different ratios (T/MDSC 1:0, 2:1, 4:1,8:1) and stimulated with ConA (5 μg/mL); unstimulated T-cells were used as a negative control. T-cell proliferation was evaluated using CFSE staining. Representative flow cytometry data **(C)** and statistical results **(D)** are shown (n=4). Neutrophils and PMN-MDSCs were extracted from the spleen of the mice treated with PBS or BQ123, and following experiments were performed. **(E)** mRNA expression level of MDSC targets, β- actin was used for normalization, and the lowest expression level sample in neutrophils group was artificially set to 1. Typical results from three experiments are shown (n=4). **(F)** Protein expression level of S100A9, Arg1, and COX2. Typical results from three experiments are shown. **(G, H)** The amount of S100A9 (G, n=9) and Arginase1 (H, n=6) in cell lysates. **(I)** To block the molecular effector of PMN-MDSCs, arginase inhibitor (NOHA, 100 μM), EP2 inhibitor AH6809 (5 μM), EP4 inhibitor L161982 (5 μM), or ROS inhibitor N-Acetylcysteine (NAC, 1 mM) was added to T/MDSC 2:1 of each group separately. Statistical results **(I)** of the frequency are shown (n=3). Data represent the mean ± SEM; *P < 0.05; **P < 0.01; ***P < 0.001; ****P < 0.0005, and ns, not significant, based on the Mann-Whitney test **(B, H)** or the two-tailed unpaired Student’s t test **(D, E, G, I)**.

BQ123 has been studied extensively with regard to its effects on endothelins (6, 7), but its effects on immune cells involved in regulating inflammation, such as MDSCs, is unclear. To explore whether BQ123 influences MDSC cell expansion in mice, we first determined the cell phenotype of MDSCs in WT mice treated with or without BQ123 injection (**Supplementary Figure 2A**) using flow cytometry. Following treatment with BQ123, the frequency of CD11b^+^Ly6G^+^Ly6C^Low/-^ cells was substantially higher in the spleen, PBMCs, and liver, but not in the bone marrow (**Figures 1A, B**). However, the frequency of CD11b^+^Ly6G^-^Ly6C^High^ cells was not affected by BQ123 (**Figures 1A, B**). This finding suggests that treatment with BQ123 increased the population of CD11b^+^Ly6G^+^Ly6C^Low/-^, but not of CD11b^+^Ly6G^-^Ly6C^High^. Considering BQ123-specific effects on PMN-MDSC expansion, the populations of other immune cells, including DCs, macrophages, and T and B cells, were also evaluated under BQ123 treatment. The data showed no change in the population of the above investigated cells (**Supplementary Figure 2B**). Hence, BQ123 revealed specific effects on PMN-MDSC expansion. Hence, BQ123 revealed specific effects on PMN-MDSC expansion.

It has been established that CD11b^+^Ly6G^+^Ly6C^Low/-^ cells in WT mice are neutrophils that do not suppress T-cell suppression (15, 16). To determine whether CD11b^+^Ly6G^+^Ly6C^Low/-^ cells stimulated by BQ123 have different biological roles in neutrophils, a T-cell proliferation assay was performed. Notably, CD11b^+^Ly6G^+^Ly6C^Low/-^ cells stimulated by BQ123 were immunosuppressive in a concentration-dependent manner (**Figures 1C, D**). However, there were no differences when compared with the control cells from mice injected with PBS (**Figures 1C, D**). It has been established that PMN-MDSCs express higher levels of S100A8 and S100A9 and lower levels of IRF8 than neutrophils (15, 16, 18). To verify the molecular biological characteristics of CD11b^+^Ly6G^+^Ly6C^Low/-^ cells stimulated by BQ123, mRNA levels of MDSC-related target genes in PMN-MDSC-like cells stimulated by BQ123 were tested. The results revealed that S100A8 and S100A9 mRNA levels were remarkably higher, IRF8 mRNA levels were significantly lower, and arg1 expression increased significantly in cells from mice treated with BQ123 when compared with those from control mice (**Figure 1E**). However, there were no differences in the mRNA levels of cyclooxygenase 2 (COX2), nicotinamide adenine dinucleotide phosphate oxidase 2 (NOX2), and inducible nitric oxide synthase (iNOS) following BQ123 treatment (**Figure 1E**). The protein levels of Arg1 and S100A9 visibly increased following BS123 treatment. This was consistent with the mRNA levels of the BQ123 treated group, as determined by western blotting and enzyme-linked immunosorbent assay (**Figures 1F–H**). However, there were no significant differences in ROS levels, COX2 protein levels, or the production of PGE2 (**Supplementary Figures 2B, C**). Additionally, CD115 (macrophage colony-stimulating factor receptor) and CD244 were used to distinguish PMN-MDSCs from neutrophils in this study (**Supplementary Figure 2E**). Hence, these data indicated that the PMN-MDSC-like cells induced by BQ123 were PMN-MDSCs, with immunosuppressive activities, evoked by MDSC-related effectors. The results also suggest that Arg1 may be mechanistically involved in the immunosuppressive effects of BQ123-induced PMN-MDSCs. To investigate whether Arg1 was required for BQ123 to regulate the activation of PMN-MDSCs, T-cell proliferation assays were performed using inhibitors of MDSC effector molecules such as NOHA (an inhibitor of arginase), NAC (an inhibitor of ROS), AH6809 (an EP2 inhibitor), and L161982 (an EP4 inhibitor). The arginase inhibitor NOHA dramatically abrogated the immunosuppressive activity of BQ123-induced PMN-MDSCs, but NAC, AH6809, and L161982 did not affect CD4^+^ T cells and CD8^+^ T cell proliferation (**Figure 1I**, **Supplementary Figure 2D**). Taken together, these results indicate that BQ123 can activate PMN-MDSCs but not M-MDSCs in mice, and that Arg1 is involved in BQ123-induced PMN-MDSC-mediated immunosuppression. Previous studies have reported that the effect of BQ123 on various diseases depends on the ET1-ETAR system (10–12, 14). To better understand the nature of the BQ123-derived PMN-MDSC, we performed additional experiments. The resultant data revealed that ETAR is expressed at high levels in PMN-MDSCs when compared with neutrophils, but the results from the ETBR were in contrast (**Supplementary Figure 2G**). Subsequent experiments revealed no obvious expansion of PMN-MDSCs after ET1 treatment (**Supplementary Figure 2H**). This suggests that BQ123, an ETAR antagonist, directly acts on MDSCs *in vivo*.

### BQ123 Activated PMN-MDSCs Through the IL13/STAT6/ARG1 Signaling Pathway

**Figure 2:**
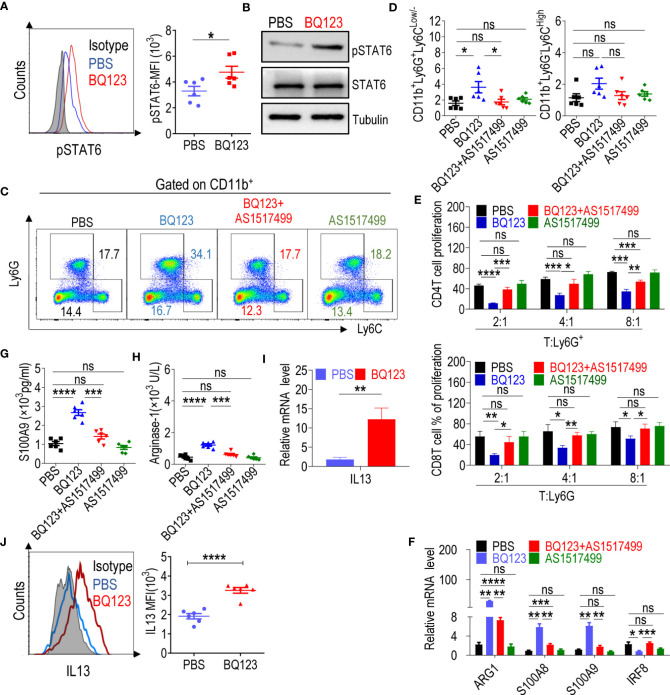
BQ123 Activated PMN-MDSCs through the IL13/STAT6/ARG1 signaling pathway. **(A)** The mean fluorescence intensity (MFI) ratio of STAT6 phosphorylation level (n=6) and **(B)** western blot of STAT6 phosphorylation level. For western blot, the typical results from three experiments are shown. Tubulin and total STAT6 were used as loading controls. For the STAT6 inhibitor experiment, mice were split into four groups. One group received PBS injections, the second group received BQ123 injections, the third group received a combination of BQ123 and AS1517499, and the fourth group received only AS1517499 injections. Ly6G^+^cells were sorted among four groups and used to complete the experiment C-F. Typical example of flow cytometry **(C)** and statistical results **(D)** from multiple experiments of the cell phenotype in spleen are shown (n=6). **(E)** A T-cell proliferation function assay was used to evaluate MDSC suppressive activity in the STAT6 inhibitor experiment. Statistical results of the frequency are shown (n=3). **(F)** mRNA expression level of the target gene including Arg1, S100A9, S100A8, and IRF8 (n=4). **(G, H)** The amount of S100A9 **(G)** and Arginase-1 **(H)** in cell lysates (n=6). **(I)** mRNA expression level of IL-13 was tested in Ly6G^+^ cells from BQ123 or PBS treated mice. β- actin was used for normalization, and the lowest expression level sample in PBS group was artificially set to 1 (n=4). **(J)** Representative flow cytometry results (left) and statistical analysis (right) of the mean fluorescence intensity (MFI) levels of IL-13 were tested in CD11b^+^Ly6G^+^Ly6C^Low/-^ cells from BQ123 or PBS treated mice (n=6). Data represent the mean ± SEM; *P < 0.05; **P < 0.01; ***P < 0.001; ****P < 0.0005, and ns, not significant, based on the Mann-Whitney test **(F, I)** or two-tailed unpaired Student’s t test **(A, D, E, G, H, J)**.

Several previous studies have shown that activation of STAT or NF-κB is linked to the suppressive activity of MDSCs (16, 18–20). Therefore, we investigated whether the transcription factors STAT1, STAT3, STAT5, STAT6, and NF-κB regulated BQ123-induced PMN-MDSC-mediated immunosuppression. To explore this question, we examined the expression of the transcription factors listed above and found that the phosphorylation level of STAT6 (pSTAT6) was significantly higher in the PMN-MDSCs from the mice that received BQ123 treatment than in those from the control group (**Figures 2A, B**). However, there were no differences in the phosphorylation levels of STAT1, STAT3, STAT5, or NF-κB (**Supplementary Figures 3A–E**). To confirm whether STAT6 is involved in BQ123 activation of PMN-MDSCs, the STAT6 inhibitor AS1517499 was used (**Supplementary Figure 4A**). As noted above, BQ123 significantly increased PMN-MDSC expansion (**Figures 2C, D**) and activated PMN-MDSC suppression (**Supplementary Figure 4B**, **Figure 2E**), leading to higher of S100A8, S100A9, and Arg1 mRNA and protein levels (**Figures 2F–H**) than those in the control group.

Treatment with BQ123 combined with AS1517499 significantly reduced BQ123 induced PMN-MDSC expansion (**Figures 2C, D**). Furthermore, treatment with BQ123 combined with AS1517499 dramatically abrogated immunosuppressive activity and recovered T-cell proliferation when compared with that in the BQ123 group (**Supplementary Figure 4B**, **Figure 2E**). Treatment with BQ123 combined with AS1517499 also led to decreased S100A8, S100A9, and Arg1 mRNA levels and increased IRF8 mRNA levels compared with those in the BQ123 group (**Figure 2F**). Finally, we tested the protein levels of MDSC molecular effectors. Treatment with BQ123 combined with AS1517499 resulted in lower levels of S100A9 and Arg1, when compared to the BQ123 group (**Figures 2G, H**). This indicated that AS1517499 reversed the effect of BQ123 on MDSC-related target genes. However, AS1517499 had no effect on the proportion of PMN-MDSCs (**Figures 2C, D**), proliferation of T-cells (**Supplementary Figure 4B**, **Figure 2E**), or the expression of MDSC molecular effectors (**Figures 2F–H**) when compared with that in the PBS control group. Thus, we propose that the inhibition of STAT6 reduced BQ123-induced PMN-MDSC-mediated immunosuppression and downregulated BQ123 induced Arg1 expression in PMN-MDSCs. Overall, BQ123 appeared to regulate PMN-MDSC immunosuppressive functions and expansion through the STAT6/Arg1 signaling pathway.

To investigate the mechanism by which BQ123-derived PMN-MDSCs induce STAT6 phosphorylation, we focused on major mechanisms previously reporting that STAT6 is usually phosphorylated by IL-4 or IL-13 (52–54). As expected, IL13 was significantly upregulated in BQ123-derived PMN-MDSCs, at both the mRNA and protein levels (**Figures 2I, J**); however, IL4 and IL5 failed to display any effects (**Supplementary Figure 4C**). These findings indicate that the activation of PMN-MDSCs triggered by BQ123 sequentially caused the upregulation of IL13, and then phosphorylated STAT6, resulting in increased Arg1 production. To determine whether BQ123 affects the production of IL13 by other cells, we performed *in vitro* ILC differentiation assays and measured the production of IL-5 and IL-13 after 5 days of treatment with BQ123. Data showed both concentrations of IL-5 and IL-13 in the supernatants and the proportion of IL5^+^ IL13^+^ ILC2s, as well as concentrations of IL-5 and IL-13 in the supernatants, was dramatically increased after BQ123 treatment, compared with the control (**Supplementary Figures 4F, 4G**), but IL5^+^IL13^+^ILC2 cells were significantly decreased. These results indicate that BQ123 potentially affected the cytokine production of ILC2.

### Both BQ123 and BQ123-Induced PMN-MDSCs Attenuated DSS-Induced Acute Colitis

**Figure 3:**
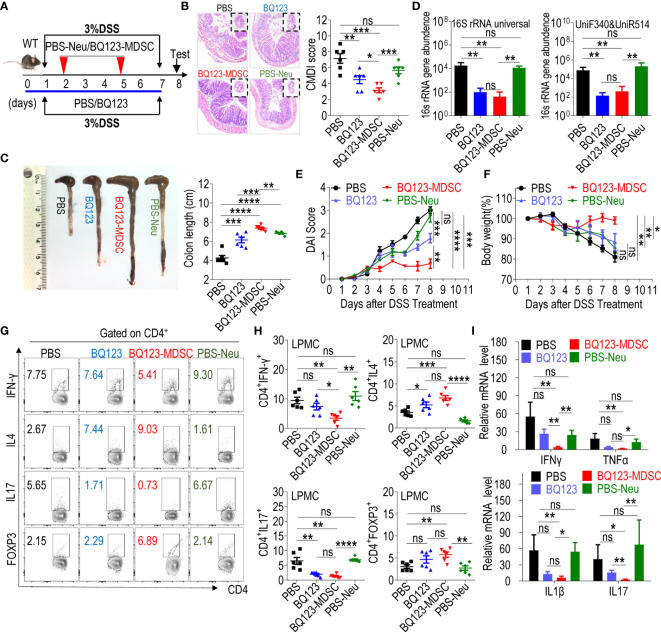
Both BQ123 and BQ123-induced PMN-MDSCs attenuate DSS-induced acute colitis. C57BL/6 male mice (six-weeks old) were divided into four groups: PBS group (DSS+PBS), BQ123 group (DSS+BQ123), BQ123-MDSC group (DSS+ BQ123-derived PMN-MDSCs), and PBS-Neu group (DSS+PBS-derived neutrophils), and fed with 3% DSS freely for seven consecutive days in the same cage. The BQ123 group was injected with 5 mg/kg for eight consecutive days, and the same route was used for the PBS group. Transfer group, about 2 × 10^6^ enriched BQ123-MDSCs (BQ123-derived PMN-MDSCs) or PBS-Neu (PBS-derived neutrophils) were injected into recipient mice on days 2 and 5. **(A)** Experimental schema. **(B)** Representative H&E staining and inflammation scores (n=6). **(C–F)** Mice were euthanized on day 8 for evaluation of colitis severity, based on: **(C)** colon length (n=6), **(D)** Bacterial loading (n=6), **(E)** Disease activity index (DAI) (n=6), and **(F)** Body weight (n=6). **(G, H)** T-cell subsets present in colon lamina propria mononuclear cells (LPMCs). A typical example of staining **(G)** and statistical results of the frequency of T-cells **(H)** (n=6) and **(I)** mRNA expression levels of inflammatory factors are shown. β-actin was used for normalization, and the lowest expression level sample in BQ123-MDSC group was artificially set to 1 (n=5). Data represent the mean ± SEM; *P < 0.05; **P < 0.01; ***P < 0.001; ****P < 0.0005, and ns, not significant, based on the Mann-Whitney test **(D, F, H)** or two-tailed unpaired Student’s t-test **(B, C, E, I)**.

As described above, BQ123 increased the suppressive activity acquisition of PMN-MDSCs *via* the STAT6/Arg1 signaling pathway. The ETAR antagonist atrasentan has been reported to control colitis *via* the ET1-ETAR system (14). To test whether BQ123 prevents the progression of acute colitis, we performed a DSS-induced acute colitis experiment (**Figure 3A**). In mice treated with BQ123, the colon inflammation and CMDI score (**Figure 3B**), shortening of colon length (**Figure 3C**), bacterial loading (**Figure 3D**), and DAI (**Figure 3E**) were significantly lower than those in the PBS group. Mice in the BQ123 group also lost slightly less body weight compared with those in the PBS group (**Figure 3F**). Moreover, PMN-MDSCs accumulated in the spleen (**Supplementary Figure 5A**) and colon (**Supplementary Figure 5B**) in the BQ123 group mice. These data suggest that BQ123 attenuates acute colitis through PMN-MDSC expansion.

Our previous study indicated that MDSC play a valuable role in controlling necrotizing enterocolitis (NEC), the most common acute gastrointestinal inflammatory emergency in preterm infants (24, 44). To investigate whether BQ123-induced PMN-MDSCs attenuate acute colitis, BQ123-induced PMN-MDSCs (BQ123-MDSC group) or neutrophils from PBS-treated mice (PBS-Neu group) were injected into WT mice through adoptive transfer using intravenous injections during DSS drinking (**Figure 3A**). After two transfers with BQ123-MDSCs, a significant remission in colon inflammation severity (**Figure 3B**), a remarkable attenuation in colon length (**Figure 3C**), less bacterial loading (**Figure 3D**), lower DAI scores (**Figure 3E**), and a noticeable alleviation of body weight loss (**Figure 3F**), were observed when compared with the PBS-Neu group. The mice in the BQ123-MDSC group appeared to be healthier than those in the BQ123 group (**Figures 3B, C, E, F**). However, the two treatments had similar effects on the bacterial loading (**Figure 3D**).

The mRNA levels of inflammatory factors in pathological colon tissues were also tested. Following treatment, a dramatic decrease in IL17, IFNγ, TNFα, and IL1β was observed in the BQ123-MDSC group but not in the PBS-Neu group (**Figure 3G**). Treatment with BQ123 appeared to slightly reduce the mRNA levels of the inflammatory factors described above, but not significantly (**Figure 3G**). Furthermore, significant reductions in IL17 and IFNγ mRNA levels were observed in the BQ123-MDSCs group, compared with the BQ123 group. Finally, T-cell subsets in the colon and spleen were measured using flow cytometry. The proportion of Th17 cells in the colon of mice from both the BQ123 and BQ123-MDSC groups was lower than that of their counterparts. Meanwhile, the proportion of Th2 cells in the colon of mice in the BQ123 and BQ123-MDSC groups was higher than that in their counterparts (**Figures 3H, I**). BQ123-MDSC transfer reduced the Th1 population and increased the Treg population compared with the PBS-Neu treatment. However, the same relationship was not observed when BQ123 was injected (**Figures 3H, I**). These results indicate that both BQ123 and BQ123-MDSC attenuated DSS-induced acute colitis. However, treatment with BQ123-induced PMN-MDSCs was more effective at relieving colitis inflammation in WT mice than treatment with BQ123 intraperitoneal injection.

### Both BQ123 and BQ123-Induced PMN-MDSCs Attenuated Papain-Induced Acute Pneumonia

**Figure 4:**
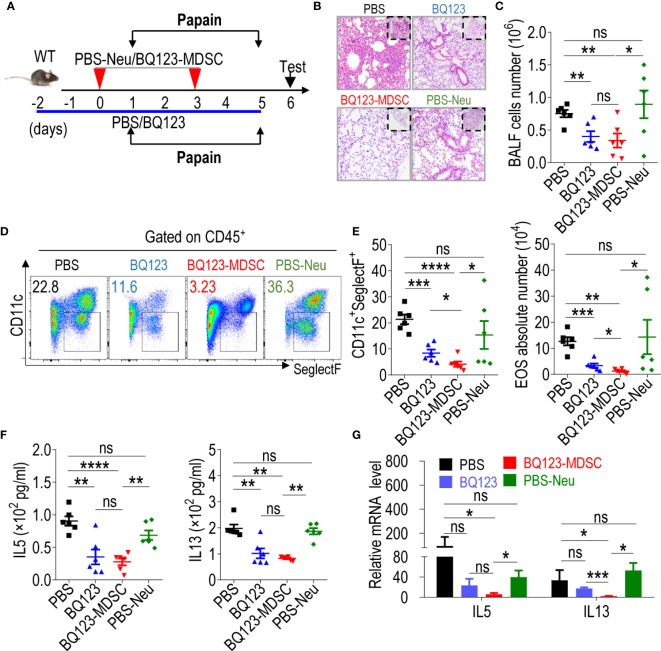
Both BQ123 and BQ123-induced PMN-MDSCs attenuate papain-induced acute pneumonia. C57BL/6 female mice (six-weeks old) were divided into four groups as shown in **Figure 3**: PBS group (papain+ PBS), BQ123 group (papain+ BQ123), BQ123-MDSC group (papain+ BQ123-derived PMN-MDSCs), and PBS-Neu group (papain+ PBS-derived neutrophils), and intranasal papain (20 µg papain in 40 µL PBS, daily) for five consecutive days in the same cage. The BQ123 group was injected with 5 mg/kg for eight consecutive days, and PBS was used as control. Transfer group, about 2 × 10^6^ enriched BQ123-MDSCs (BQ123-derived PMN-MDSCs) or PBS-Neu (PBS-derived neutrophils) were injected into recipient mice on days 0 and 3. **(A)** Experimental design. **(B)** Representative H&E staining of lung sections. **(C)** Absolute number of BALF (n=6). **(D)** Typical example of flow cytometry and **(E)** statistical results both population and the absolute number of EOS in the BALF (n=6). **(F)** The level of IL-5 and IL-13 in the BALF samples (n=6). **(G)** The mRNA expression levels of inflammatory factors, we used β- actin for normalization, and the lowest expression level sample in BQ123-MDSC group was artificially set to 1 (n=4). Data represent the mean ± SEM; *P < 0.05; **P < 0.01; ***P < 0.001; ****P < 0.0005, and ns, not significant, based on the Mann-Whitney test **(E, G)** or two-tailed unpaired Student’s t test **(C, F)**.

BQ123 has been reported to reduce fibrocyte differentiation and progression in chronic obstructive asthma, a type of persistent airway inflammation (13). We investigated whether BQ123-and BQ123-induced PMN-MDSCs have a similar effect on acute pneumonia inflammation. To explore this question, acute pneumonia was induced using papain through intranasal instillation. Pneumonia mice were divided into four groups: PBS, BQ123, BQ123- MDSC, and PBS-Neu, as shown in **Figure 4A**. Histological evidence showed that lung inflammation was lower in mice that received a BQ123 injection than in those that had received PBS (**Figure 4B**). We also found that the total BALF number, as well as the influx and frequency of eosinophils (EOS) in the BALF (**Figures 4C–E**) were significantly lower in the BQ123 group than in the PBS group. As expected, lower levels of IL5 and IL13 were produced in the BALF of the BQ123 group than in the PBS group (**Figure 4F**). Furthermore, the mRNA levels of IL5 and IL13 in the lung tissue of the BQ123 group were slightly lower than those in the PBS group (**Figure 4G**). PMN-MDSCs were also accumulated in the spleen (**Supplementary Figure 6A**) and lungs (**Supplementary Figure 6B**) of the mice in the BQ123 group. These results suggest that BQ123 attenuates acute pneumonia with PMN-MDSC expansion.

Inflammation was remarkably lower in the lungs of mice that received a transfer of two PMN-MDSCs than in the lungs of mice in the PBS-Neu group (**Figures 4B, E**). The total number of cells (**Figure 4C**) and influx of eosinophils in the BALF of the BQ123-MDSC group was significantly lower than that in the PBS-Neu group (**Figures 4D, E**). Furthermore, IL5 and IL13 production was lower (**Figure 4F**) in the BALF of the BQ123-MDSC group than in the PBS-Neu group. Finally, the levels of IL5 and IL13 mRNA in the lungs of the BQ123 and BQ123-MDSC groups were significantly lower than those in the BALF of the PBS-Neu group (**Figure 4G**). These data suggest that BQ123-induced PMN-MDSCs also attenuate acute pneumonia.

Interestingly, the severity of fibrosis and inflammatory hyperplasia in the alveolus was reduced to a greater extent in the BQ123-MDSC group than in the BQ123 group (**Figures 4B–E**). More importantly, the influx and frequency of eosinophils in the BALF were significantly lower in mice treated with BQ123-induced PMN-MDSCs than in those treated with BQ123 (**Figures 4D, E**). However, there were no significant differences in the levels of inflammatory cytokines in the BALF (**Figure 4F**). Furthermore, the mRNA level of IL13 in the BQ123-MDSC group was remarkably lower than that in the BQ123 group (**Figure 4G**). Together with inflammation remission, BQ123-MDSC of the spleen and lung accumulation (**Supplementary Figures 6A, B**) were observed in the BQ123 group. These observations indicate that both BQ123 and BQ123-induced PMN-MDSCs attenuated acute lung inflammation, and that treatment with BQ123 relieved pneumonia with PMN-MDSC expansion. BQ123-induced PMN-MDSC transfer was more effective in attenuating acute pneumonia, suggesting a promising future for cellular immunotherapy.

### Both BQ123 and BQ123-Induced PMN-MDSCs Attenuated ConA-Induced Acute Hepatitis

**Figure 5:**
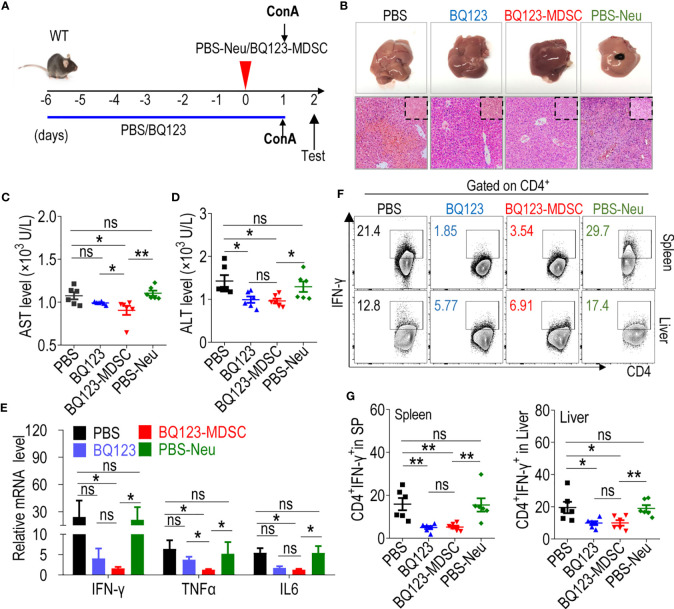
Both BQ123 and BQ123-induced PMN-MDSCs attenuate ConA-induced acute hepatitis. C57BL/6 male mice (six-weeks of age) were divided into four groups as shown in **Figure 3**: PBS group (ConA+ PBS), BQ123 group (ConA+ BQ123), BQ123-MDSC group (ConA+ BQ123-derived PMN-MDSCs), and PBS-Neu group (ConA+ PBS-derived neutrophils), and were given a single tail intravenous injection of ConA (Sigma-Aldrich) at a dose of 13 mg/kg body weight for 24 hours in the same cage. The BQ123 group was injected with 5 mg/kg for eight consecutive days, and the same route was used for the PBS group. Transfer group, about 2× 10^6^ enriched BQ123-MDSCs (BQ123-derived PMN-MDSCs) or PBS-Neu (PBS-derived neutrophils) were injected into recipient mice 1 h before receiving the ConA injection on day 0. **(A)** Experimental design. **(B)** Representative H&E staining and gross pathological appearance. **(C, D)** Levels of AST and ALT in serum (n=6). **(E)** The mRNA expression levels of inflammatory factors, β- actin was used for normalization, and the lowest expression level sample in BQ123-MDSC group was artificially set to 1 (n=4). **(F, G)** Th1 cells in the mouse SP and liver. Typical examples of flow cytometry **(F)** and statistical results of multiple experiments **(G)** are shown (n=6). Data represent mean ± SEM; *P < 0.05; **P < 0.01, and ns, not significant, based on the Mann-Whitney test **(E, G)** or two-tailed unpaired Student’s t test **(C, D)**.

We induced acute hepatitis using ConA (**Figure 5A**) to determine whether BQ123- and BQ123-induced PMN-MDSCs attenuated acute inflammation. Hematoxylin and eosin (H&E) staining revealed that mice treated with BQ123 experienced less bleeding and fewer caseous necrotic lesions than those in the PBS group, suggesting that BQ123 was more effective at alleviating liver inflammation (**Figure 5B**). The overall appearance of the liver tissue also returned to ruddy after BQ123 administration (**Figure 5B**). Of note, the levels of ALT were dramatically lower, and AST levels were slightly lower in mice treated with BQ123 than in mice administered PBS (**Figures 5C, D**). Moreover, the mRNA levels of inflammatory factors, including IFNγ, TNFα, and IL6, were also slightly lower in the BQ123 group than in the PBS group (**Figure 5E**). In addition, the levels of Th1 cells infiltrating the spleen and liver were noticeably lower in the BQ123 group than in the PBS group (**Figures 5F, G**). Similar to the effects observed for colitis and pneumonia, PMN-MDSCs accumulated in the spleen and liver following treatment with BQ123 (**Supplementary Figures 7A, B**). These results suggest that BQ123 attenuated acute hepatitis with PMN-MDSC expansion. H&E staining indicated that treatment with BQ123-MDSC alleviated liver inflammation. Furthermore, there were still many obvious caseous necrotic lesions in the PBS-Neu group (**Figure 5B**). Levels of AST and ALT in the serum were substantially lower in mice in the BQ123-MDSC transfer group than in those in the PBS-Neu transfer group (**Figures 5C, D**). Moreover, a dramatic decrease in IFNγ, TNFα, and IL6 expression was observed following BQ123-MDSC adoptive transfer when compared with the PBS-Neu treatment (**Figure 5E**). Additionally, the level of Th1 cells infiltrating the spleen and liver noticeably decreased following treatment with BQ123-MDSC, compared with the PBS-Neu treatment (**Figures 5F, G**). These results indicated that BQ123-induced PMN-MDSCs also attenuated acute hepatitis.

There were no obvious differences in the pathological alleviation between the BQ123 and BQ123-MDSC groups (**Figure 5B**). However, the reduction in AST level was more pronounced in the BQ123-MDSC group than in the BQ123 group (**Figure 5C**). Mice in the BQ123-MDSC transfer group had significantly lower levels of TNFα compared to those in the BQ123 group (**Figure 5E**). However, the Th1 population in the spleen and liver did not differ between the BQ123 and BQ123-MDSC groups (**Figures 5F, G**). These results confirmed that BQ123- and BQ123-induced PMN-MDSCs contributed to the remission of acute hepatitis inflammation. BQ123 administration relieved hepatitis with PMN-MDSC expansion, and BQ123-induced PMN-MDSC transfer was more effective in attenuating acute hepatitis.

### Depletion of PMN-MDSC Induced by BQ123 Aggravated Acute Inflammation

**Figure 6:**
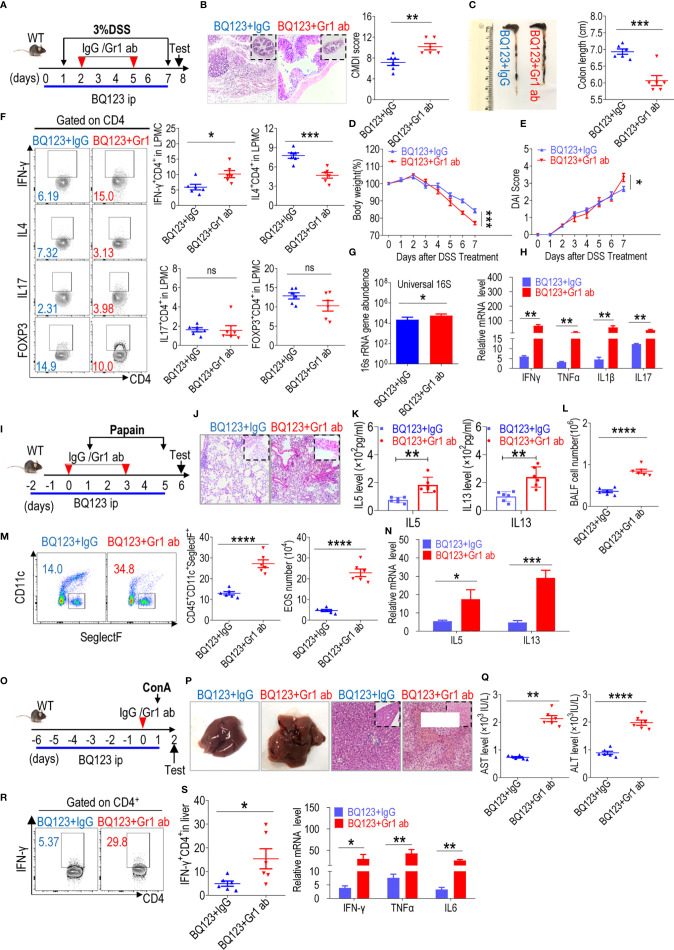
Depletion of PMN-MDSC induced by BQ123 aggravated acute inflammation. Depletion of MDSC was performed using anti-Gr1. The mice (six-weeks old) were injected with BQ123 as shown in **Figure 1** before induction model. And mice were divided into two groups: anti-lgG group (BQ123+ lgG), anti-Gr1 group (BQ123+Gr1ab). In depletion experiment, Gr1ab or IgG (10 mg/kg) was administered intraperitoneally to C57BL/6 WT mice. **(A–H)** Depletion experiment of DSS-induced acute colitis. **(A)** Experimental design, Gr1ab was injected into recipient mice on days 2 and 5. **(B)** Representative H&E staining and CMDI score (n=6), **(C)** Colon Length (n=6), **(D)** Body weight (n=6), **(E)** clinical symptom score (DAI) (n=6), **(F)** T-cell subsets present in colon lamina propria mononuclear cells (LPMCs) are shown. A typical example of staining (left) and statistical results (right) of the frequency of T-cells are analysis (n=6). **(G)** Bacterial loading (n = 5), and **(H)** the mRNA expression levels of inflammatory factors (n=5) are presented, β- actin was used for normalization, and the lowest expression level sample in BQ123+ lgG group was artificially set to 1. (I-N) Depletion experiment of papain-induced acute pneumonia. **(I)** Experimental design, Gr1ab was injected into recipient mice at days 0 and 3. **(J)** Representative H&E staining, **(K)** IL-5 and IL-13 levels in BALF (n=6) and **(L)** the absolute number of EOS in the BALF are presented (n=6), **(M)** Typical example of flow cytometry and statistical results both population and the absolute number of EOS in the BALF (n=6), and **(N)** the mRNA expression levels of inflammatory factors (n=5) are shown, β- actin was used for normalization, and the lowest expression level sample in BQ123+ lgG group was artificially set to 1. **(O–S)** Depletion experiment of ConA-induced acute hepatitis. **(O)** Experimental design, Gr1ab was injected into recipient mice 1 h before receiving the ConA injection on day 0. **(P)** Representative H&E staining and gross pathological appearance, **(Q)** levels of AST and ALT in the serum (n=6) and **(R)** Th1 cells in the mouse liver are presented. Typical examples of flow cytometry and statistical results of multiple experiments (n=6), and **(S)** the mRNA expression levels of inflammatory factors are shown, β- actin was used for normalization, and the lowest expression level sample in BQ123+ lgG group was artificially set to 1 (n=5). Data represent the mean ± SEM; *P < 0.05; **P < 0.01; ***P < 0.001; ****P < 0.0005, and ns, not significant, based on the Mann-Whitney test **(H, K, M, N, Q, R)** or two-tailed unpaired Student’s t test **(B–G, L, S)**.

In mice, targeting Gr1 by employing an agonistic antibody depleted PMN-MDSCs (55, 56). Agonistic Gr1 antibody depletion experiments were performed to determine the effect of BQ123-derived PMN-MDSC depletion on three acute inflammation cases (**Figures 6A, I, O**). After depleting PMN-MDSCs by injection with an anti-Gr1 antibody, the population of Gr1^+^CD11b^+^ MDSCs in the lamina propria, lung, or liver was significantly reduced when compared with the IgG control (**Supplementary Figures 8A–C**). Along with the decrease in Gr1^+^CD11b^+^ MDSCs, Gr1ab significantly aggravated colon inflammation severity (**Figures 6A–H**), as manifested by decreased body weight loss and increased colon inflammation score, DAI scores, intestinal permeability, bacterial loading, and inflammatory factor mRNA levels. Both Th1 and Th17 population in the colon from Gr1 ab groups were increased when compared with IgG control. Meanwhile, the proportion of Th2 and Treg cells in the colon were decreased when compared with IgG control (**Figure 6F**, **Supplementary Figure 8D**). Similar to the effects observed during colitis, increased inflammation was observed in the pneumonia model (**Figures 6I–N**), including increased lung inflammation, eosinophil frequency, total BALF cell number, absolute eosinophil number, IL5 and IL13 production in BALF, and mRNA levels of IL5 and IL13 in lung tissues. In the hepatitis model, as expected, the degree to which inflammation was aggravated was similar to that observed in the above two models (**Figures 6O–S**), including increased liver inflammatory injury, serum AST and ALT levels, and the mRNA levels of inflammatory cytokines in the tissues. Furthermore, the level of Th1 cells infiltrating the liver was noticeably higher following Gr1ab injection when compared with the isotype control (**Figure 6R**, and **Supplementary Figure 8E**). Our results showed that BQ123-induced PMN-MDSCs mediated the reduction in acute inflammation.

### BQ123-Induced PMN-MDSCs Controlled Acute Inflammation in T-Cell-Dependent Manner

**Figure 7:**
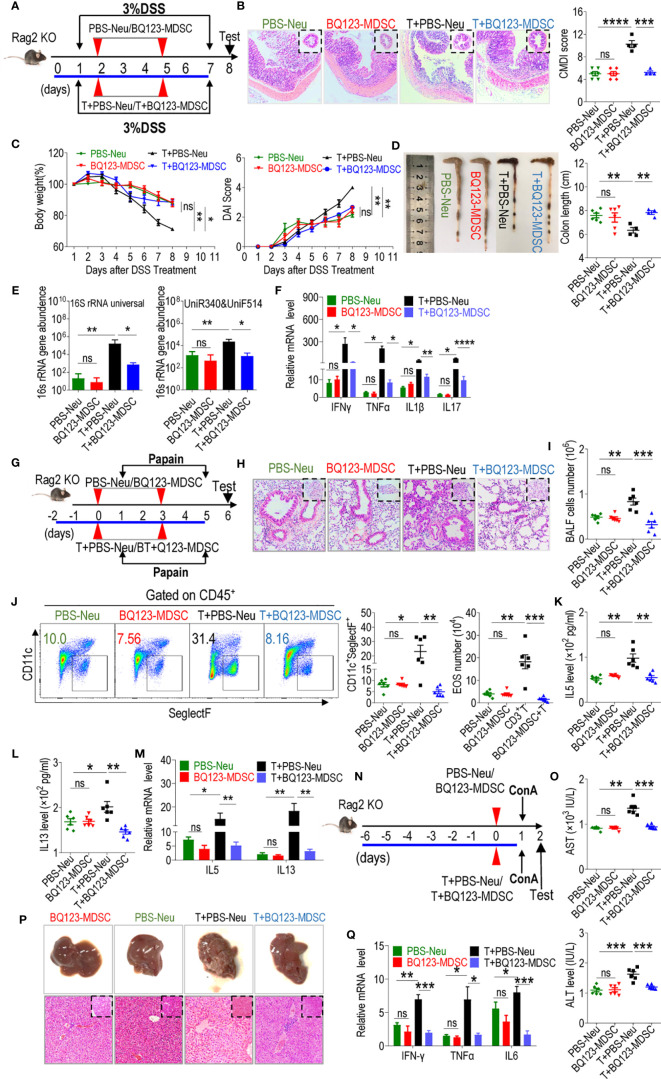
BQ123-derived PMN-MDSCs control acute inflammation in T-cell-dependent manner. Rag2 KO mice were divided in four groups, one group received neutrophils from PBS-treated mice (PBS-Neu), second group received BQ123-induced PMN-MDSCs (BQ123-MDSC), and third group is a combination of CD3^+^ T and PBS-Neu cells (T+PBS-Neu), fourth group are a combination of CD3^+^ T and BQ123-MDSC cells (T+ BQ123-MDSC). The three mouse models, as shown in **Figures 3**–**5**, respectively, were performed. About 2×10^6^ BQ123-PMN-MDSCs or PBS-Neu were intravenously injected into recipient mice on different days among four groups, for third and fourth group, about 4 × 10^6^ CD3^+^T cells from donor mice was also injected into Rag2 KO recipient mice in the same cycle. **(A–F)** DSS-induced acute colitis. **(A)** Experimental design, **(B)** Representative H&E staining, and CMDI score, **(C)** clinical symptom score (DAI) and Body weight, **(D)** Gross pathological appearance and Colon Length, **(E)** bacterial loading, and **(F)** The mRNA expression levels of inflammatory factors are shown, β- actin was used for normalization, and the lowest expression level sample in BQ123-MDSC group was artificially set to 1(n=4). (PBS-Neu group and BQ123-MDSCs group, n=6, T+PBS-Neu group, T+ BQ123-MDSC group, n=4). **(G-M)** Papain-induced acute pneumonia. **(G)** Experimental design, **(H)** Representative H&E staining, **(I)** Absolute number of BALF (n=6), **(J)** A typical example of flow cytometry and statistical results from both population and the absolute number of EOS in the BALF (All group n=6), **(K)** IL-5 and **(L)** IL-13 levels in BALF (n=6), and **(M)** The mRNA expression levels of inflammatory factors are presented, β- actin was used for normalization, and the lowest expression level sample in BQ123-MDSC group was artificially set to 1(n=5). **(N–Q)** ConA-induced acute hepatitis. **(N)** Experimental design, **(O)** Levels of AST and ALT in the serum (n=6), **(P)** Representative H&E staining and gross pathological appearance, and **(Q)** The mRNA expression levels of inflammatory factors are shown, β- actin was used for normalization, and the lowest expression level sample in BQ123-MDSC group was artificially set to 1 (n=4). Data represent the mean ± SEM; *P < 0.05; **P < 0.01; ***P < 0.001; ****P < 0.0005, and ns, not significant, based on the Mann-Whitney test **(E, F, K)** or two-tailed unpaired Student’s t test **(B–D, I, J, L, M, O, Q)**.

Several studies have demonstrated that PMN-MDSCs inhibit inflammation by suppressing over-activated T-cells (24, 44, and 52). We explored whether BQ123-induced PMN-MDSCs required T-cells to attenuate acute inflammation. Here, we utilized recombination-activating gene 2 deficient mice (Rag2 KO) with impaired T- and B-cell development to induce acute inflammation. The mice were divided into four groups: one group received neutrophils from PBS-treated mice (PBS-Neu), the second group received BQ123-induced PMN-MDSCs (BQ123-MDSC), the third group received a combination of PBS-Neu and CD3^+^T cells, and the fourth group received a combination of BQ123-MDSC and CD3^+^T cells. In the colitis model (**Figure 7A**), following adoptive transfers, no apparent differences were observed between BQ123-MDSC and PBS-Neu treatment groups in terms of colon inflammation severity (**Figures 7B–E**), including colon length, body weight loss, DAI scores, and bacterial loading. Moreover, no significant differences were detected in the mRNA levels of inflammatory factors between the two groups (**Figure 7F**); however, the transfer of PBS-Neu and CD3^+^T cells increased the inflammation index (**Figures 7B–F**). Administration of BQ123-MDSCs and CD3^+^T cells abrogated these effects (**Figures 7B–F**). In the pneumonia model using Rag2 KO mice (**Figure 7G**), the degree to which inflammation was alleviated was similar in the BQ123-MDSC and PBS-Neu groups (**Figures 7H–M**), as indicated by the severity of histological inflammation, eosinophil frequency, total BALF cell number, absolute eosinophil number, and IL5 and IL13 production in BALF, as well as the mRNA levels of IL5 and IL13. Similar to the colitis model, PBS-Neu and CD3^+^T cell transfer increased the inflammation index (**Figures 7H–M**). Administration of BQ123-MDSC and CD3^+^T cells abrogated these effects (**Figures 7H–M**). After adoptive transfer in Rag2 KO mice presenting the hepatitis model (**Figure 7N**), as expected, no obvious differences in inflammatory index were observed between the BQ123-MDSC and PBS-Neu groups (**Figures 7O–Q**), including inflammatory injury, serum AST and ALT levels, and the mRNA levels of inflammatory cytokines in liver tissues. Consistent with the above two models, the inflammation index values were increased following the co-transfer of PBS-Neu and CD3^+^T cells (**Figures 7O–Q**) and administration of BQ123-MDSC and CD3^+^T cells abrogated these effects (**Figures 7O–Q**). Our results revealed that T-cell deficiency resulted in the loss of PMN-MDSC targets for the alleviation of inflammation. BQ123-induced PMN-MDSCs reduced acute immune-mediated inflammation in a T cell-dependent manner.

## Discussion

Previous studies have reported that BQ123, a selective endothelin-A receptor (ETAR) antagonist, is widely used to test the physiological and pathophysiological roles of endothelins. It is also used to treat cardiovascular diseases such as hypertension and obesity cardiomyopathy because of the regulatory effect of endothelin on the ET1-ETAR system (4, 5, 14, 57). A few studies have reported that BQ123 suppresses the ET1-ETAR system-mediated inflammatory cytokine TNFα, preventing inflammation-induced airway smooth muscle hyperplasia and diabetic retinopathy (10, 12). BQ123 has also been found to reduce ROS production and TNF-α and IL-6 levels through its effects on the ET1-ETAR system, resulting in enhanced antioxidant defense under high glucose conditions (9). Another ETAR inhibitor, atrasentan, has also been reported to have a therapeutic role in controlling DSS-induced colitis (14). However, to the best of our knowledge, whether BQ123 can alleviate acute inflammatory disease directly, and the mechanisms by which BQ123 affects inflammation are poorly understood and require further research. In this study, we revealed that BQ123directly attenuated acute inflammatory diseases such as DSS-induced colitis, papain-induced pneumonia, and ConA-induced hepatitis. Together with pathological alleviation, the infiltrated proinflammatory cells in the tissue were decreased in mice treated with BQ123. Interestingly, inflammatory cytokines in tissue pathologies were slightly alleviated. These results suggest a new therapeutic use and novel understanding of BQ123 for ameliorating immune-mediated inflammatory diseases.

The presence of MDSCs in newborns has been reported to control the inflammatory response during early life and could be used to treat infants with NEC (24). Moreover, lactoferrin from breast milk induces PMN-MDSCs in preterm infants and has potent anti-inflammatory effects (44). In addition, atorvastatin-derived PMN-MDSCs can attenuate DSS-induced murine chronic colitis (58). In this study, we first observed ETAR expression in MDSCs, but ET1 failed to affect MDSC expansion. Subsequently, we revealed that BQ123, an ETAR inhibitor, specifically promoted PMN-MDSC activation, together with MDSC-related target genes and the molecular effectors S100A8, S100A9, and Arg1. Treatment with BQ123 relieved acute inflammation through PMN-MDSC expansion in the spleen and diseased tissue. To explore the efficacy of BQ123-induced PMN-MDSCs as a form of cellular immunotherapy, we used adoptive transfer techniques to introduce either BQ123-induced PMN-MDSCs or neutrophils from control mice into WT mice with acute colitis, acute pneumonia, and acute hepatitis. In mice with acute colitis, acute pneumonia, and acute hepatitis, we observed significant remission of inflammation. Furthermore, remarkable reductions in Th1 and Th17 population infiltration were observed in the colon with acute colitis. There were also noticeable decreases in the eosinophil population in the BALF of mice with acute pneumonia, and significant reductions in Th1 population infiltration in the liver were observed following the transfer of BQ123-induced PMN-MDSCs. Together with decreased pro-inflammatory cells, downregulated inflammatory cytokines were also observed in the three acute inflammation models. To comprehensively elucidate the specific effect of BQ123-MDSC in the above three acute inflammation models, we depleted BQ123-MDSCs in these mice using an agonistic Gr1 antibody. Gr1ab significantly worsened the inflammation index; however, no impact was observed when treated with IgG isotype control. These results indicate that BQ123 facilitated PMN-MDSC expansion and activation. Furthermore, BQ123-derived PMN-MDSCs truly mediated the attenuation of acute inflammatory disease, proposing a promising future for cellular immunotherapy.

T-cells are essential for mediating adaptive immunity in response to a variety of pathogens, especially acute inflammation. Furthermore, infiltrated T-cells are considered particularly important for pathological progression (59, 60). Our previous studies have indicated that targeting T-cells is key to alleviating inflammatory diseases (24, 44, 58). Other studies have also suggested that MDSCs suppress inflammation by inhibiting the over-activation of T-cells (61, 62). Consistent with the published literature, we also demonstrated that adoptive transfer with BQ123-induced PMN-MDSCs can alleviate acute inflammation in the presence of T-cells. To explore the specificity of BQ123-induced PMN-MDSC targeting T-cells in alleviating acute inflammation, T-cell-deficient mice (Rag2 KO mice) were used. As expected, neither BQ123-MDSC nor PBS-Neu transfer into Rag2 KO mice affected the severity of acute inflammation or inflammatory cytokine levels. Administration of CD3-T cells and PBS-Neu aggravated acute inflammation. In striking contrast, co-injection of BQ123-MDSC and CD3-T cells ameliorated the inflammation index. Therefore, we propose that BQ123-induced PMN-MDSCs alleviate acute inflammation in a T cell-dependent manner. Next, we assessed whether BQ123 promoted PMN-MDSC expansion and activation in a T-cell-manner using Rag2 KO mice. Following BQ123 injection, we observed that the PMN-MDSC population was enhanced, as well as showed suppression activity, in Rag2 KO mice (**Supplementary Figures 9A, B**). Therefore, we concluded that BQ123-MDSC alleviated acute inflammation in a T-cell-manner, with no significant impact on PMN-MDSC activation in T cell-deficient mice.

In summary, our results identified BQ123 as a potential anti-inflammatory drug, controlling acute immune relative inflammatory diseases such as DSS-induced colitis, papain-induced pneumonia, and ConA-induced hepatitis through PMN-MDSC activation. Moreover, BQ123 activated PMN-MDSCs through the IL-13/STAT6/Arg1 signaling pathway. In addition to injections of BQ123, the adoptive transfer of BQ123-induced PMN-MDSCs (BQ123-MDSCs) also significantly attenuated acute immune inflammatory disease in the presence of T-cells. In contrast, BQ123-MDSCs lost their targets and were effective in the absence of T-cells. Thus, BQ123-MDSCs appear to control acute inflammation in a T cell-dependent manner. Further research into BQ123-MDSCs may provide a new avenue for cellular immunotherapy in inflammatory diseases.

## Data Availability Statement

The datasets presented in this study can be found in online repositories. The names of the repository/repositories and accession number(s) can be found in the article/**Supplementary Material**.

## Ethics Statement

The study was reviewed and approved by the Medical Ethics Committee of the Southern Medical University. All animal experiments in this study were approved by the Welfare and Ethical Committee for Experimental Animal Care of the Southern Medical University. Written informed consent was obtained from the owners for the participation of their animals in this study.

## Author Contributions

YH, JL, and GX conceived and supervised the study and wrote the manuscript. ZC, XZ, SL, ZX, MS, XL, MC, SZ, and YT performed the experiments. Authors with equal contributions are listed in alphabetical order. All authors contributed to the article and approved the submitted version.

## Funding

This work was supported by grants from the following institutions: the High-level Talent Start-up Funding of the Southern Medical University, the National Natural Science Foundation of China (31700061, 81971420, and 81991511), the Guangdong Special Support Program for Youth Science and Technology Innovation Talents (2019TQ05Y585), the National Natural Science Foundation of Guangdong (2019A1515011435), and the Science and Technology Program of Guangzhou (201904010073).

## Conflict of Interest

The authors declare that the research was conducted in the absence of any commercial or financial relationships that could be construed as a potential conflict of interest.

## References

[1] Diehl KJ Stauffer BL Dow CA Bammert TD Brunjes DL Greiner JJ. Chronic Nebivolol Treatment Suppresses Endothelin-1-Mediated Vasoconstrictor Tone in Adults with Elevated Blood Pressure. Hypertension (2016) 67:1196–204. 10.1161/HYPERTENSIONAHA.115.06979 PMC4871319 27113048

[2] Dhaun N Webb DJ. Endothelins in cardiovascular biology and therapeutics. Nat Rev Cardiol (2019) 16:491–502. 10.1038/s41569-019-0176-3 30867577

[3] Gupta RM Hadaya J Trehan A Zekavat SM Roselli C Klarin D. A Genetic Variant Associated with Five Vascular Diseases Is a Distal Regulator of Endothelin-1 Gene Expression. Cell (2017) 170:522–33. 10.1016/j.cell.2017.06.049 PMC5785707 28753427

[4] Guo Q Tian Y Wang Z Li A Ma Z Guo Y. Endothelin receptors in augmented vasoconstrictor responses to endothelin-1 in chronic intermittent hypoxia. Clin Exp Pharmacol Physiol (2013) 40:449–57. 10.1111/1440-1681.12109 23662699

[5] Ceylan-Isik AF Dong M Zhang Y Dong F Turdi S Nair S. Cardiomyocyte-specific deletion of endothelin receptor A rescues aging-associated cardiac hypertrophy and contractile dysfunction: role of autophagy. Basic Res Cardiol (2013) 108:335. 10.1007/s00395-013-0335-3 23381122 PMC3590116

[6] Briyal S Gulati A. Endothelin-A receptor antagonist BQ123 potentiates acetaminophen induced hypothermia and reduces infarction following focal cerebral ischemia in rats. Eur J Pharmacol (2010) 644:73–9. 10.1016/j.ejphar.2010.06.071 20638381

[7] Schulze-Neick I Li J Reader JA Shekerdemian L Redington AN Penny DJ. The endothelin antagonist BQ123 reduces pulmonary vascular resistance after surgical intervention for congenital heart disease. J Thorac Cardiovasc Surg (2002) 124:435–41. 10.1067/mtc.2002.121492 12202858

[8] Yeager ME Belchenko DD Nguyen CM Colvin KL Ivy DD Stenmark KR. Endothelin-1, the unfolded protein response, and persistent inflammation: role of pulmonary artery smooth muscle cells. Am J Respir Cell Mol Biol (2012) 46:14–22. 10.1165/rcmb.2010-0506OC 21778413 PMC3262656

[9] Kowalczyk A Kleniewska P Kolodziejczyk M Skibska B Goraca A. The role of endothelin-1 and endothelin receptor antagonists in inflammatory response and sepsis. Arch Immunol Ther Exp (2015) 63:41–52. 10.1007/s00005-014-0310-1 PMC4289534 25288367

[10] Alrashdi SF Deliyanti D Wilkinson-Berka JL. Intravitreal administration of endothelin type A receptor or endothelin type B receptor antagonists attenuates hypertensive and diabetic retinopathy in rats. Exp Eye Res (2018) 176:1–9. 10.1016/j.exer.2018.06.025 29944850

[11] Fouda MA Abdel-Rahman AA. Endothelin Confers Protection against High Glucose-Induced Neurotoxicity *via* Alleviation of Oxidative Stress. J Pharmacol Exp Ther (2017) 361:130–9. 10.1124/jpet.116.238659 PMC5363775 28179472

[12] Knobloch J Yanik SD Korber S Stoelben E Jungck D Koch A. TNFalpha-induced airway smooth muscle cell proliferation depends on endothelin receptor signaling, GM-CSF and IL-6. Biochem Pharmacol (2016) 116:188–99. 10.1016/j.bcp.2016.07.008 27422754

[13] Weng C Chen B Wang C Feng P Lee M Huang C. The endothelin A receptor mediates fibrocyte differentiation in chronic obstructive asthma. The involvement of connective tissue growth factor. Am J Respir Crit Care Med (2013) 188:298–308. 10.1164/rccm.201301-0132OC 23795584

[14] Claudino RF Leite DF Bento AF Chichorro JG Calixto JB Rae GA. Potential role for ET-2 acting through ETA receptors in experimental colitis in mice. Inflammation Res (2017) 66:141–55. 10.1007/s00011-016-1001-7 27778057

[15] Veglia F Perego M Gabrilovich D. Myeloid-derived suppressor cells coming of age. Nat Immunol (2018) 19:108–19. 10.1038/s41590-017-0022-x PMC5854158 29348500

[16] Bronte V Brandau S Chen SH Colombo MP Frey AB Greten TF. Recommendations for myeloid-derived suppressor cell nomenclature and characterization standards. Nat Commun (2016) 7:12150. 10.1038/ncomms12150 27381735 PMC4935811

[17] Marvel D Gabrilovich DI. Myeloid-derived suppressor cells in the tumor microenvironment: expect the unexpected. J Clin Invest (2015) 125:3356–64. 10.1172/JCI80005 PMC4588239 26168215

[18] Zhou J Nefedova Y Lei A Gabrilovich D. Neutrophils and PMN-MDSC: Their biological role and interaction with stromal cells. Semin Immunol (2018) 35:19–28. 10.1016/j.smim.2017.12.004 29254756 PMC5866202

[19] Kumar V Patel S Tcyganov E Gabrilovich DI. The Nature of Myeloid-Derived Suppressor Cells in the Tumor Microenvironment. Trends Immunol (2016) 37:208–20. 10.1016/j.it.2016.01.004 PMC4775398 26858199

[20] Veglia F Tyurin VA Blasi M De Leo A Kossenkov AV Donthireddy L. Fatty acid transport protein 2 reprograms neutrophils in cancer. Nature (2019) 569:73–8. 10.1038/s41586-019-1118-2 PMC6557120 30996346

[21] Yang Q Li X Chen H Cao Y Xiao Q He Y. IRF7 regulates the development of granulocytic myeloid-derived suppressor cells through S100A9 transrepression in cancer. Oncogene (2017) 36:2969–80. 10.1038/onc.2016.448 28092673

[22] Dorhoi A Du Plessis N. Monocytic Myeloid-Derived Suppressor Cells in Chronic Infections. Front Immunol (2017) 8:1895. 10.3389/fimmu.2017.01895 29354120 PMC5758551

[23] Pawelec G Verschoor CP Ostrand-Rosenberg S. Myeloid-Derived Suppressor Cells: Not Only in Tumor Immunity. Front Immunol (2019) 10:1099. 10.3389/fimmu.2019.01099 31156644 PMC6529572

[24] He Y Li X Perego M Nefedova Y Kossenkov AV Jensen EA. Transitory presence of myeloid-derived suppressor cells in neonates is critical for control of inflammation. Nat Med (2018) 24:224–31. 10.1038/nm.4467 PMC5803434 29334374

[25] Ulas T Pirr S Fehlhaber B Bickes MS Loof TG Vogl T. S100-alarmin-induced innate immune programming protects newborn infants from sepsis. Nat Immunol (2017) 18:622–32. 10.1038/ni.3745 28459433

[26] Zhao A Xu H Kang X Zhao A Lu L. New insights into myeloid-derived suppressor cells and their roles in feto-maternal immune cross-talk. J Reprod Immunol (2016) 113:35–41. 10.1016/j.jri.2015.11.001 26599285

[27] Pan T Liu Y Zhong L Shi M Duan X Wu K. Myeloid-derived suppressor cells are essential for maintaining feto-maternal immunotolerance *via* STAT3 signaling in mice. J Leukoc Biol (2016) 100:499–511. 10.1189/jlb.1A1015-481RR 27203698

[28] Li M Zhu D Wang T Xia X Tian J Wang S. Roles of Myeloid-Derived Suppressor Cell Subpopulations in Autoimmune Arthritis. Front Immunol (2018) 9:2849. 10.3389/fimmu.2018.02849 30564242 PMC6288996

[29] Deshane JS Redden DT Zeng M Spell ML Zmijewski JW Anderson JT. Subsets of airway myeloid-derived regulatory cells distinguish mild asthma from chronic obstructive pulmonary disease. J Allergy Clin Immunol (2015) 135:413–24. 10.1016/j.jaci.2014.08.040 PMC4323991 25420684

[30] Knier B Hiltensperger M Sie C Aly L Lepennetier G Engleitner T. Myeloid-derived suppressor cells control B cell accumulation in the central nervous system during autoimmunity. Nat Immunol (2018) 19:1341–51. 10.1038/s41590-018-0237-5 PMC6241855 30374128

[31] Elliott DM Singh N Nagarkatti M Nagarkatti PS. Cannabidiol Attenuates Experimental Autoimmune Encephalomyelitis Model of Multiple Sclerosis Through Induction of Myeloid-Derived Suppressor Cells. Front Immunol (2018) 9:1782. 10.3389/fimmu.2018.01782 30123217 PMC6085417

[32] Taams LS. Inflammation and immune resolution. Clin Exp Immunol (2018) 193:1–2. 10.1111/cei.13155 29987840 PMC6037995

[33] Salas JR Chen BY Wong A Cheng D Van Arnam JS Witte ON. 18F-FAC PET Selectively Images Liver-Infiltrating CD4 and CD8 T Cells in a Mouse Model of Autoimmune Hepatitis. J Nucl Med (2018) 59:1616–23. 10.2967/jnumed.118.210328 PMC6167535 29700125

[34] Xu C Zhang C Ji J Wang C Yang J Geng B. CD36 deficiency attenuates immune-mediated hepatitis in mice by modulating the proapoptotic effects of CXC chemokine ligand 10. Hepatology (2018) 67:1943–55. 10.1002/hep.29716 29220536

[35] Feng X Chi G Wang H Gao Y Chen Q Ru Y. IL-37 suppresses the sustained hepatic IFN-gamma/TNF-alpha production and T cell-dependent liver injury. Int Immunopharmacol (2019) 69:184–93. 10.1016/j.intimp.2019.01.037 30735937

[36] Sang X Wang R Zhang C Liu S Shen H Guo Y. Sophocarpine Protects Mice from ConA-Induced Hepatitis *via* Inhibition of the IFN-Gamma/STAT1 Pathway. Front Pharmacol (2017) 8:140. 10.3389/fphar.2017.00140 28377718 PMC5359249

[37] Ritter B Greten FR. Modulating inflammation for cancer therapy. J Exp Med (2019) 216:1234–43. 10.1084/jem.20181739 PMC6547855 31023715

[38] Crusz SM Balkwill FR. Inflammation and cancer: advances and new agents. Nat Rev Clin Oncol (2015) 12:584–96. 10.1038/nrclinonc.2015.105 26122183

[39] Greten FR Grivennikov SI. Inflammation and Cancer. Triggers, Mechanisms, and Consequences. Immunity (2019) 51:27–41. 10.1016/j.immuni.2019.06.025 31315034 PMC6831096

[40] White AA Stevenson DD. Aspirin-Exacerbated Respiratory Disease. N Engl J Med (2018) 379:1060–70. 10.1056/NEJMra1712125 30207919

[41] Vandewalle J Luypaert A De Bosscher K Libert C. Therapeutic Mechanisms of Glucocorticoids. Trends Endocrinol Metab (2018) 29:42–54. 10.1016/j.tem.2017.10.010 29162310

[42] Dinarello CA. Interleukin-1 in the pathogenesis and treatment of inflammatory diseases. Blood (2011) 117:3720–32. 10.1182/blood-2010-07-273417 PMC3083294 21304099

[43] McIntyre LA Stewart DJ Mei SHJ Courtman D Watpool I Granton J. Cellular Immunotherapy for Septic Shock. A Phase I Clinical Trial. Am J Respir Crit Care Med (2018) 197:337–47. 10.1164/rccm.201705-1006OC 28960096

[44] Liu Y Perego M Xiao Q He Y Fu S He J. Lactoferrin-induced myeloid-derived suppressor cell therapy attenuates pathologic inflammatory conditions in newborn mice. J Clin Invest (2019) 129:4261–75. 10.1172/JCI128164 PMC6763238 31483289

[45] Monticelli LA Buck MD Flamar AL Saenz SA Tait Wojno ED Yudanin NA. Arginase 1 is an innate lymphoid-cell-intrinsic metabolic checkpoint controlling type 2 inflammation. Nat Immunol (2016) 17:656–65. 10.1038/ni.3421 PMC4873382 27043409

[46] He Y Chen Y Song W Zhu L Dong Z. Ow DW. A Pap1-Oxs1 signaling pathway for disulfide stress in Schizosaccharomyces pombe. Nucleic Acids Res (2017) 45:106–14. 10.1093/nar/gkw818 PMC5224502 27664222

[47] Wirtz S Popp V Kindermann M Gerlach K Weigmann B Fichtner-Feigl S. Chemically induced mouse models of acute and chronic intestinal inflammation. Nat Protoc (2017) 12:1295–309. 10.1038/nprot.2017.044 28569761

[48] Yang JY Kim MS Kim E Cheon JH Lee YS Kim Y. Enteric Viruses Ameliorate Gut Inflammation *via* Toll-like Receptor 3 and Toll-like Receptor 7-Mediated Interferon-beta Production. Immunity (2016) 44:889–900. 10.1016/j.immuni.2016.03.009 27084119

[49] Proietti M Cornacchione V Rezzonico Jost T Romagnani A Faliti CE Perruzza L. ATP-gated ionotropic P2X7 receptor controls follicular T helper cell numbers in Peyer’s patches to promote host-microbiota mutualism. Immunity (2014) 41:789–801. 10.1016/j.immuni.2014.10.010 25464855

[50] Barman M Unold D Shifley K Amir E Hung K Bos N. Enteric salmonellosis disrupts the microbial ecology of the murine gastrointestinal tract. Infect Immun (2008) 76:907–15. 10.1128/IAI.01432-07 PMC2258829 18160481

[51] Arshad MI Piquet-Pellorce C L’Helgoualc’h A Rauch M Patrat-Delon S Ezan F. TRAIL but not FasL and TNF-alpha, regulates IL-33 expression in murine hepatocytes during acute hepatitis. Hepatology (2012) 56:2353–62. 10.1002/hep.25893 22961755

[52] Lin Y Li B Yang X Liu T Shi T Deng B. Non-hematopoietic STAT6 induces epithelial tight junction dysfunction and promotes intestinal inflammation and tumorigenesis. Mucosal Immunol (2019) 12:1304–15. 10.1038/s41385-019-0204-y 31534167

[53] Liang Y Yi P Yuan D Jie Z Kwota Z Soong L. IL-33 induces immunosuppressive neutrophils *via a* type 2 innate lymphoid cell/IL-13/STAT6 axis and protects the liver against injury in LCMV infection-induced viral hepatitis. Cell Mol Immunol (2019) 16:126–37. 10.1038/cmi.2017.147 PMC6355846 29400707

[54] Su S Zhao Q He C Huang D Liu J Chen F. miR-142-5p and miR-130a-3p are regulated by IL-4 and IL-13 and control profibrogenic macrophage program. Nat Commun (2015) 6:8523. 10.1038/ncomms9523 26436920 PMC4600756

[55] Baert T Vankerckhoven A Riva M Van Hoylandt A Thirion G Holger G. Myeloid Derived Suppressor Cells: Key Drivers of Immunosuppression in Ovarian Cancer. Front Immunol (2019) 10:1273. 10.3389/fimmu.2019.01273 31214202 PMC6558014

[56] Boivin G Faget J Ancey PB Gkasti A Mussard J Engblom C. Durable and controlled depletion of neutrophils in mice. Nat Commun (2020) 11:2762. 10.1038/s41467-020-16596-9 32488020 PMC7265525

[57] Lakhal-Littleton S Crosby A Frise MC Mohammad G Carr CA Loick PAM. Intracellular iron deficiency in pulmonary arterial smooth muscle cells induces pulmonary arterial hypertension in mice. Proc Natl Acad Sci USA (2019) 116:13122–30. 10.1073/pnas.1822010116 PMC6600981 31152133

[58] Lei A Yang Q Li X Chen H Shi M Xiao Q. Atorvastatin promotes the expansion of myeloid-derived suppressor cells and attenuates murine colitis. Immunology (2016) 149:432–46. 10.1111/imm.12662 PMC5095490 27548304

[59] Bertin S Aoki-Nonaka Y Lee J de Jong PR Kim P Han T. The TRPA1 ion channel is expressed in CD4(+) T cells and restrains T-cell-mediated colitis through inhibition of TRPV1. Gut (2017) 66:1584–96. 10.1136/gutjnl-2015-310710 PMC5173457 27325418

[60] Chen L He Z Iuga AC Martins Filho SN Faith JJ Clemente JC. Diet Modifies Colonic Microbiota and CD4(+) T-Cell Repertoire to Induce Flares of Colitis in Mice with Myeloid-Cell Expression of Interleukin 23. Gastroenterology (2018) 155:1177–91. 10.1053/j.gastro.2018.06.034 PMC6174107 29909020

[61] Rieber N Singh A Oz H Carevic M Bouzani M Amich J. Pathogenic fungi regulate immunity by inducing neutrophilic myeloid-derived suppressor cells. Cell Host Microbe (2015) 17:507–14. 10.1016/j.chom.2015.02.007 PMC4400268 25771792

[62] Lian M Wang Q Jiang X Zhang J Wei Y Li Y. The Immunobiology of Receptor Activator for Nuclear Factor Kappa B Ligand and Myeloid-Derived Suppressor Cell Activation in Immunoglobulin G4-Related Sclerosing Cholangitis. Hepatology (2018) 68:1922–36. 10.1002/hep.30095 29774578

