# Supplementary material for: Treatment With Endothelin-A Receptor Antagonist BQ123 Attenuates Acute Inflammation in Mice Through T-Cell-Dependent Polymorphonuclear Myeloid-Derived Suppressor Cell Activation
Source: Front Immunol. 2021 Mar 22;12:641874. doi: 10.3389/fimmu.2021.641874 (PMC8019801; doi:10.3389/fimmu.2021.641874)
Supplement: Supplementary file 1 [file DataSheet_1.pdf]

Sup Figure 1

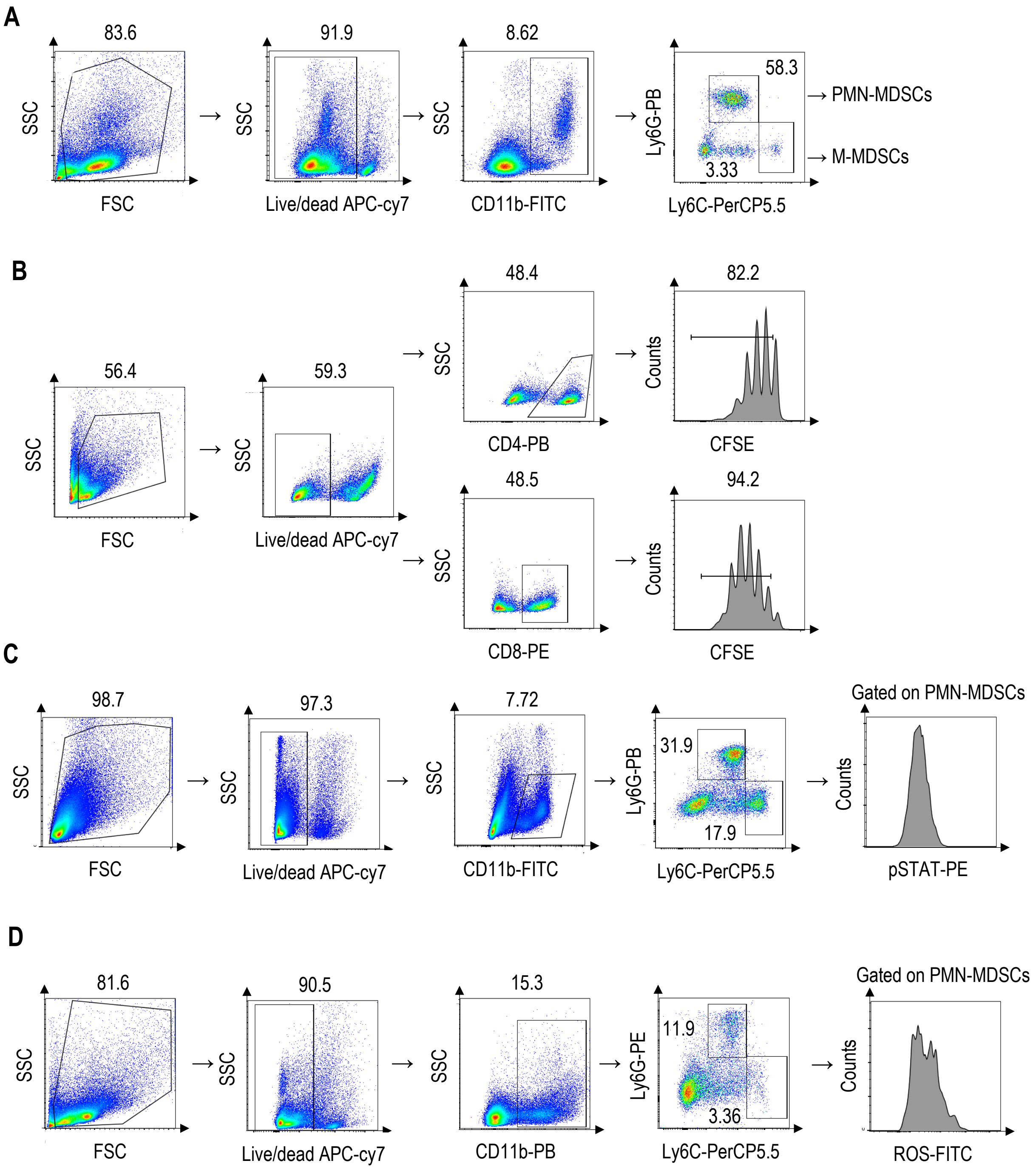

Supplementary Figure 1. Gating strategy for flow cytometry

Gating strategy for MDSC phenotype (A), T- cell suppression activity (B), phosphorylation level of STAT family (C) and ROS level (D).

Sup Figure 2

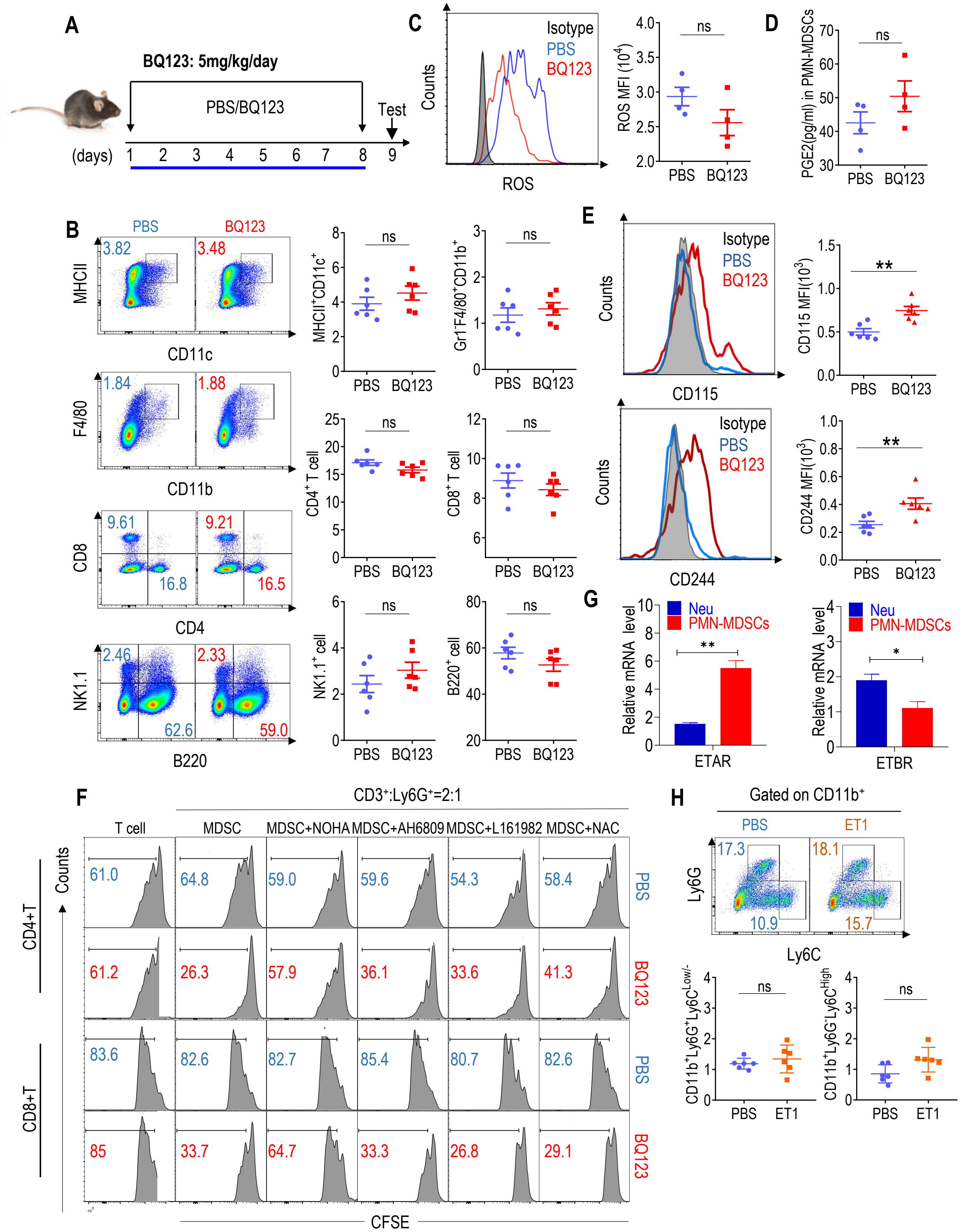

A
BQ123: 5mg/kg/day
PBS/BQ123
Test
(days) 1 2 3 4 5 6 7 8 9
C
Isotype
PBS
BQ123
Counts
ROS
ROS MFI (10⁴)
ns
PBS BQ123
D
PGE2(pg/ml) in PMN-MDSCs
ns
PBS BQ123
B
PBS
BQ123
3.82
3.48
MHCII
CD11c
1.84
1.88
F4/80
CD11b
9.61
9.21
16.8
16.5
CD8
CD4
2.46
2.33
62.6
59.0
NK1.1
B220
MHCII⁺CD11c⁺
Gr1⁻F4/80⁺CD11b⁺
CD4⁺ T cell
CD8⁺ T cell
NK1.1⁺ cell
B220⁺ cell
ns
PBS BQ123
E
Isotype
PBS
BQ123
Counts
CD115
CD115 MFI(10³)
CD244
CD244 MFI(10³)
**
G
Neu
PMN-MDSCs
Relative mRNA level
ETAR
ETBR
**
*
F
CD3⁺:Ly6G⁺=2:1
T cell
MDSC
MDSC+NOHA
MDSC+AH6809
MDSC+L161982
MDSC+NAC
Counts
CD4+T
CD8+T
61.0 64.8 59.0 59.6 54.3 58.4
PBS
61.2 26.3 57.9 36.1 33.6 41.3
BQ123
83.6 82.6 82.7 85.4 80.7 82.6
PBS
85 33.7 64.7 33.3 26.8 29.1
BQ123
CFSE
H
Gated on CD11b⁺
PBS
ET1
17.3
18.1
10.9
15.7
Ly6G
Ly6C
CD11b⁺Ly6G⁺Ly6C^Low/-
CD11b⁺Ly6G⁻Ly6C^High
ns
PBS ET1

Supplementary Figure 2. Arg1 Inhibitor NOHA Reversed the Immunosuppressive Function of PMN-MDSCs Induced by BQ123.

Four-week-old C57BL/6 mice were injected intraperitoneally with BQ123 at a dose of 5uM/kg for eight consecutive days. Controls received PBS via the same route. Mice were euthanized for analysis 24 h after the last administration. (A) Experimental scheme development. (B) The proportions of DC ($MHCII^+CD11c^+$), macrophage ($Gr1^-F4/80^+CD11b^+$), NK ($NK1.1^+$), T($CD4^+CD8^+$), and B cell ($B220^+$) was measured, and representative flow cytometry results (left) and statistical analysis (right) are shown. (n=6). (C) Representative flow cytometry results and statistical analysis of ROS of neutrophils from mice treated with PBS and PMN-MDSC from mice treated with BQ123 (n=4). (D) PGE2 levels in PMN-MDSCs and control cells (n=4). (E) Representative flow cytometry results (left) and statistical analysis (right) of the mean fluorescence intensity (MFI) levels of CD115 and CD244in $CD11b^+Ly6G^+Ly6C^{low/-}$ cells from PBS group and BQ123 group were evaluated by flow cytometry. Representative flow cytometry results (left) and statistical analysis (right) are shown.(n=6). (F) Representative flow cytometry results of the T cell proliferation with MDSC effectors inhibitors.(G) mRNA expression level of ETAR and ETBR in neutrophils and PMN-MDSCs were evaluated by qRT-PCR. β-actin was used for normalization, and the lowest expression level sample in neutrophils group was artificially set to 1 (n=3). (H) Four-week-old C57BL/6 mice were injected intraperitoneally with ET-1 at a dose of 5mg/kg for eight consecutive days. PBS was used as control. Mice were euthanized for analysis 24 h after the last administration. The proportions of MDSC subsets in the spleen was measured using flow cytometry analysis. A typical example of flow cytometry and statistical results from multiple experiments are shown (n=6). Data represent mean ± SEM; $*P < 0.05$; $**P < 0.01$, and ns= not significant, using two-tailed unpaired Student's t test (B, C, D ,and G) or the Mann-Whitney test (E, H) .

Sup Figure 3

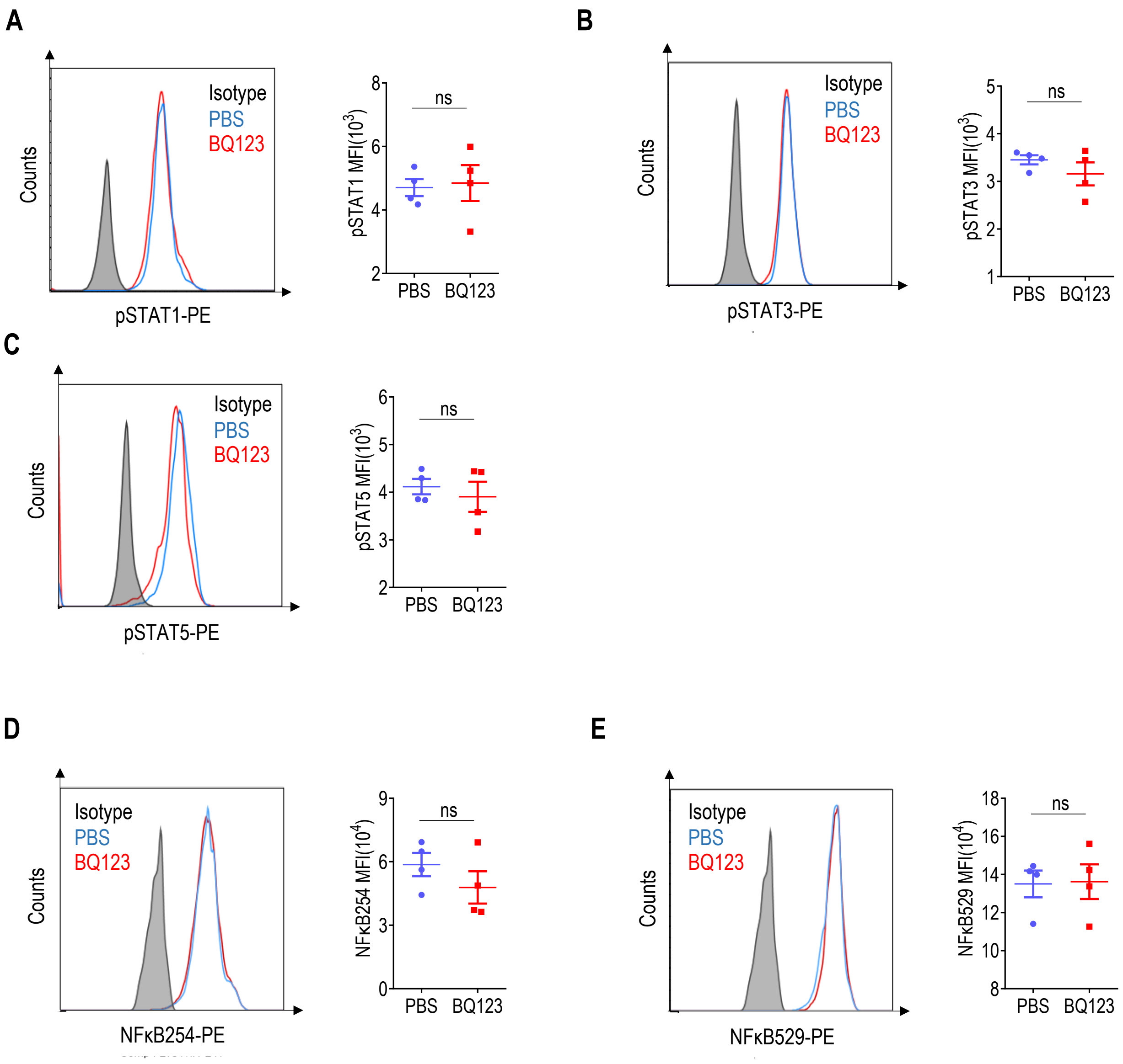

Supplementary Figure 3. The Upstream Phosphorylation Level

Representative flow cytometry results (left) and statistical analysis (right) of the mean fluorescence intensity (MFI) levels of p-STAT1(A), p-STAT3 (B), p-STAT5 (C), and NF-κβ254/529 (D, E) in PMN-MDSCs and control cells. Data represent the mean ± SEM; *P < 0.05; **P < 0.01; ***P < 0.001, and ns= not significant, and are based on two-tailed unpaired Student's t test (A, B, C, D and E).

Sup Figure 4

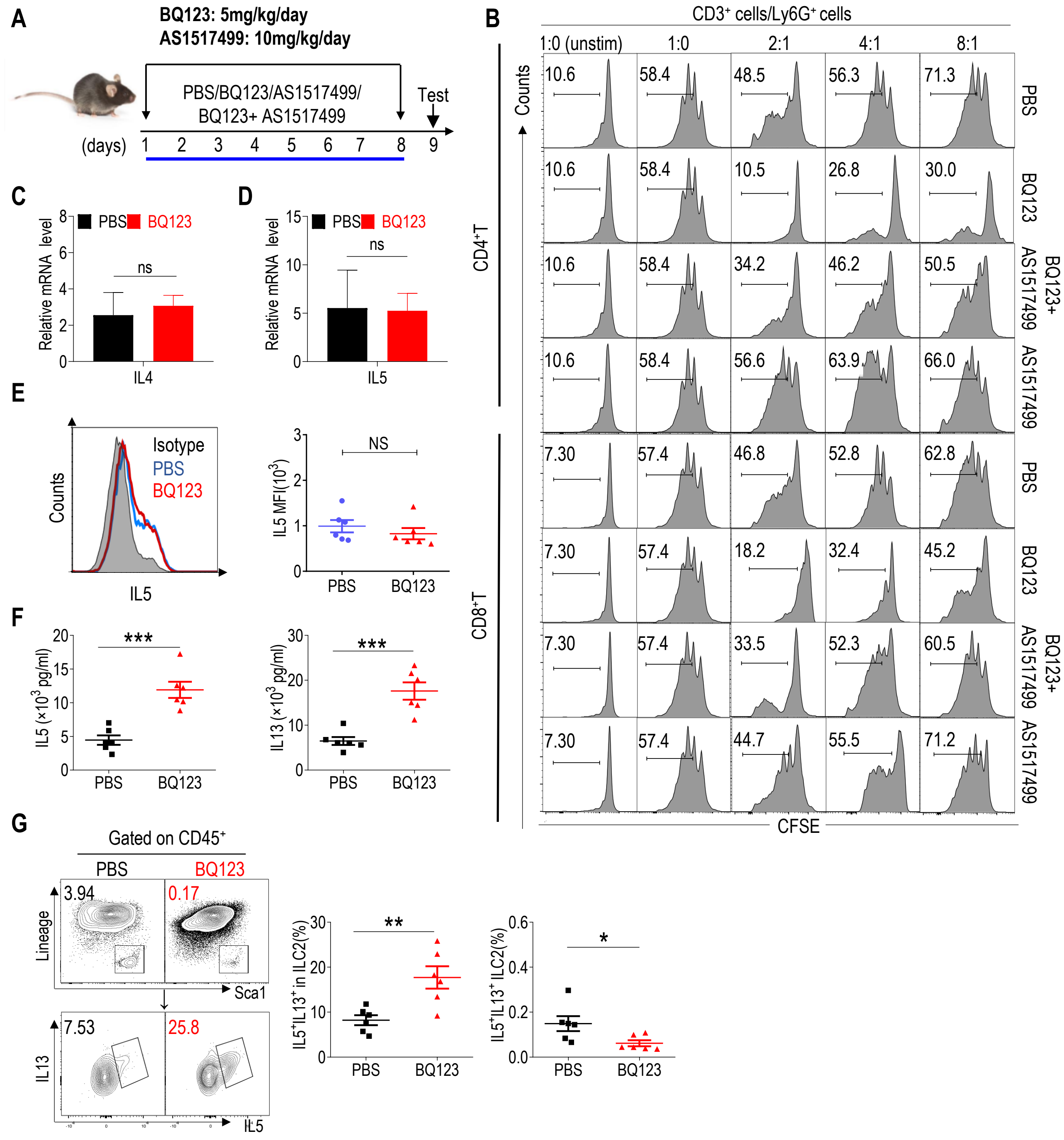

A
BQ123: 5mg/kg/day
AS1517499: 10mg/kg/day
PBS/BQ123/AS1517499/
BQ123+ AS1517499
Test
(days) 1 2 3 4 5 6 7 8 9
B
CD3+ cells/Ly6G+ cells
1:0 (unstim) 1:0 2:1 4:1 8:1
Counts
CFSE
CD4+T
CD8+T
PBS
BQ123
BQ123+ AS1517499
AS1517499
10.6 58.4 48.5 56.3 71.3
10.6 58.4 10.5 26.8 30.0
10.6 58.4 34.2 46.2 50.5
10.6 58.4 56.6 63.9 66.0
7.30 57.4 46.8 52.8 62.8
7.30 57.4 18.2 32.4 45.2
7.30 57.4 33.5 52.3 60.5
7.30 57.4 44.7 55.5 71.2
C
PBS BQ123
Relative mRNA level
ns
IL4
D
PBS BQ123
Relative mRNA level
ns
IL5
E
Isotype
PBS
BQ123
Counts
IL5
IL5 MFI(10³)
NS
PBS BQ123
F
IL5 (×10³ pg/ml)
***
PBS BQ123
IL13 (×10³ pg/ml)
***
PBS BQ123
G
Gated on CD45+
PBS BQ123
3.94 0.17
Lineage
Sca1
7.53 25.8
IL13
IL5
IL5+IL13+ in ILC2(%)
**
PBS BQ123
IL5+IL13+ ILC2(%)
*
PBS BQ123

Supplementary Figure 4. STAT6 Inhibitor AS1517499 Abrogated the Function of PMN-MDSCs Activated by BQ123

C57BL/6 male mice (six-weeks of age) were divided into four : One group received PBS injections, the second group received BQ123 injections, the third group received a combination of BQ123 and AS1517499, and the fourth group received only AS1517499 injections. (A) Experimental design. (B) Same to Figure 1C, T-cell proliferation function assay was used to evaluate MDSC suppressive activity from mice with or without STAT6 inhibitor. Representative flow cytometry data are shown. (C-D) mRNA expression level of IL-4 and IL-5 in neutrophils and PMN-MDSCs were evaluated by qRT-PCR. We used β- actin for normalization, and the lowest expression level sample in control group was artificially set to 1 (n=5). (E) Representative flow cytometry results (left) and statistical analysis (right) of the mean fluorescence intensity (MFI) protein levels of IL-5.(n=6). (F) The amounts of IL-5 and IL-13 in co-culture supernatants were evaluated by ELISA (n=6). (G) Sorted lineage^{-} cells from bone marrow (BM) co-culture with BQ123 were cultured within the presence of interleukin-2 (IL-2) (20 ng/ml), IL-7 (20 ng/ml) and IL-33 (100ng/ml) for 5 days. For the secretion of cytokines from ILC2s, ILC2s were stimulated for 4 hr with brefeldin A, PMA, and ionomycin. Representative flow cytometric and statistical results of Lin^{-}Sca1^{+} ILC2 cells and IL-5^{+} IL-13^{+} in ILC2s was showed(n=6). Data represent mean ± SEM; *P < 0.05; **P < 0.01; ***P < 0.001, and ns= not significant, using two-tailed unpaired Student's t test (F and G) or the Mann-Whitney test (C , D ,and E) .

Sup Figure 5

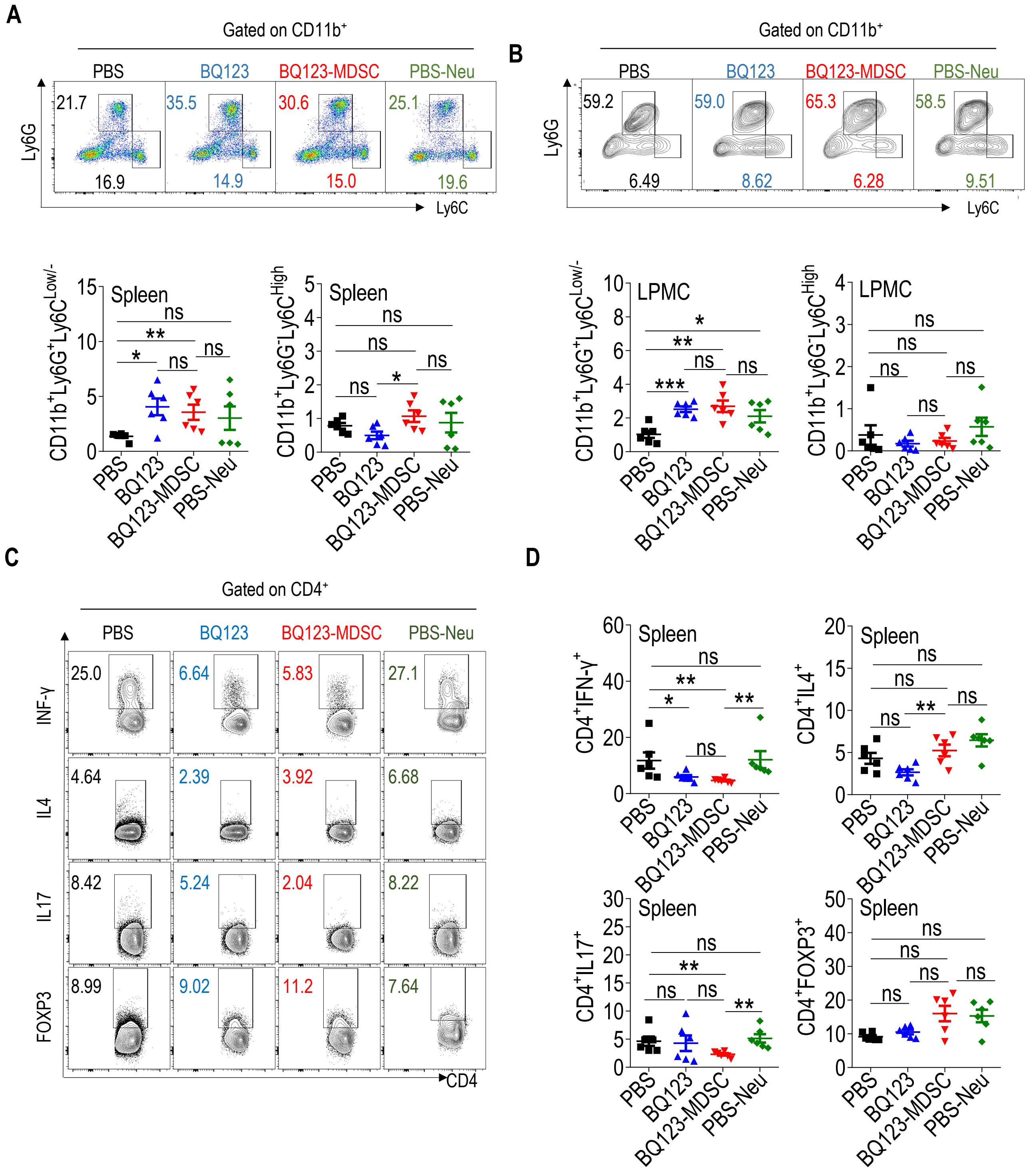

Supplementary Figure 5. Both BQ123 and BQ123-induced PMN-MDSCs Attenuate DSS-Induced Acute Colitis

(A and B) Phenotypes of MDSCs in the SP (A) and LPMC (B). Representative flow cytometry results (upper) and statistical analysis (lower) are shown (n=6). (C-D) T cell subsets in the SP. Representative flow cytometry results (C) and statistical analysis (D) are shown (n=6). Data represent the mean ± SEM; *$P < 0.05$; **$P < 0.01$, and ns= not significant, and are based on the Mann-Whitney test (D) or two-tailed unpaired Student's t test (A, B).

Sup Figure 6

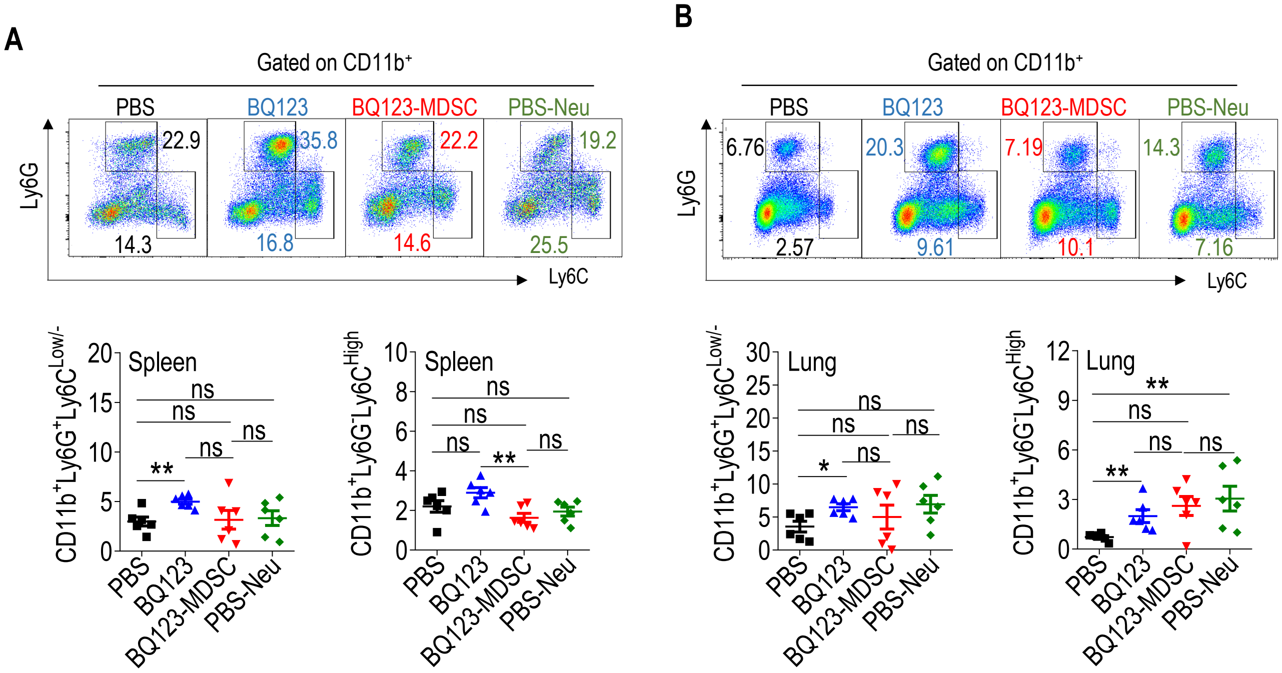

Supplementary Figure 6. Both BQ123 and BQ123-induced PMN-MDSCs Relieve Papain-Induced Acute Pneumonia

(A and B) Phenotype of MDSCs in the SP (A) and lung (B). Representative flow cytometry results (upper) and statistical analysis (lower) are shown (n=6). Data represent the mean ± SEM; *P < 0.05; **P < 0.01, and ns= not significant, and are based on the two-tailed unpaired Student's t test (A, B).

Sup Figure 7

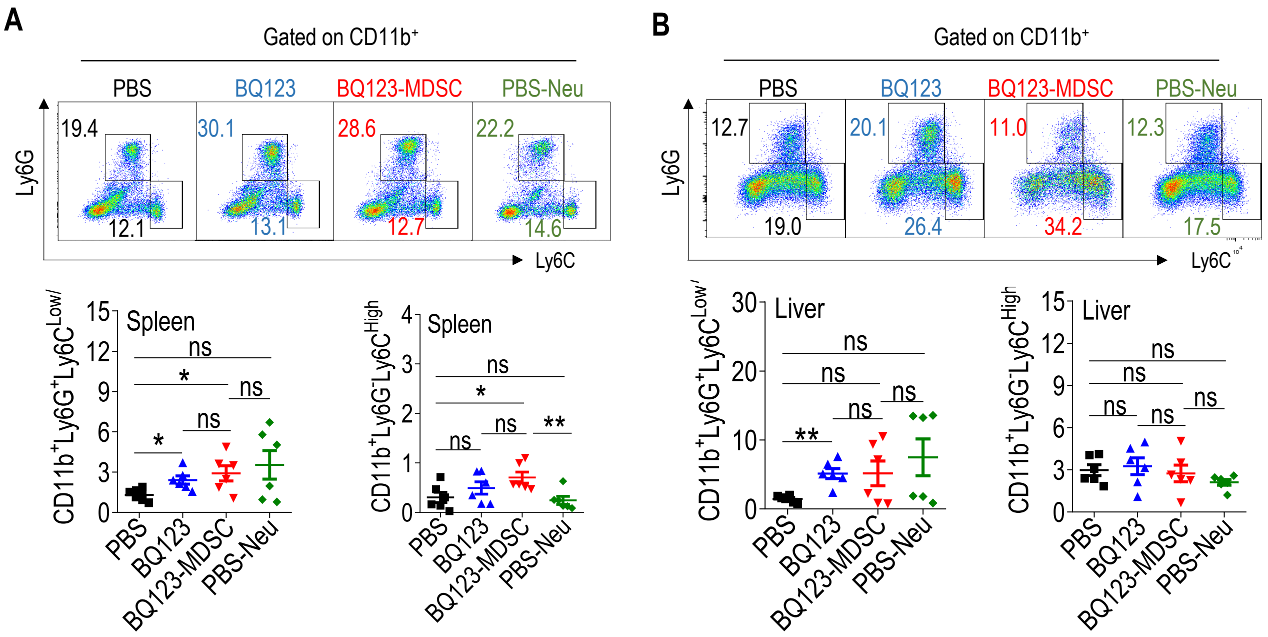

Supplementary Figure 7. BQ123 and BQ123-induced PMN-MDSCs Alleviated ConA-Induced Murine Hepatitis

(A and B) Phenotypes of MDSCs in the SP (A) and liver (B). Representative flow cytometry results (upper) and statistical analysis (lower) are shown (n=6). Data represent the mean ± SEM; *P < 0.05; **P < 0.01, and ns= not significant, and are based on the Mann-Whitney test (B) or the two-tailed unpaired Student's t test (A).

Sup Figure 8

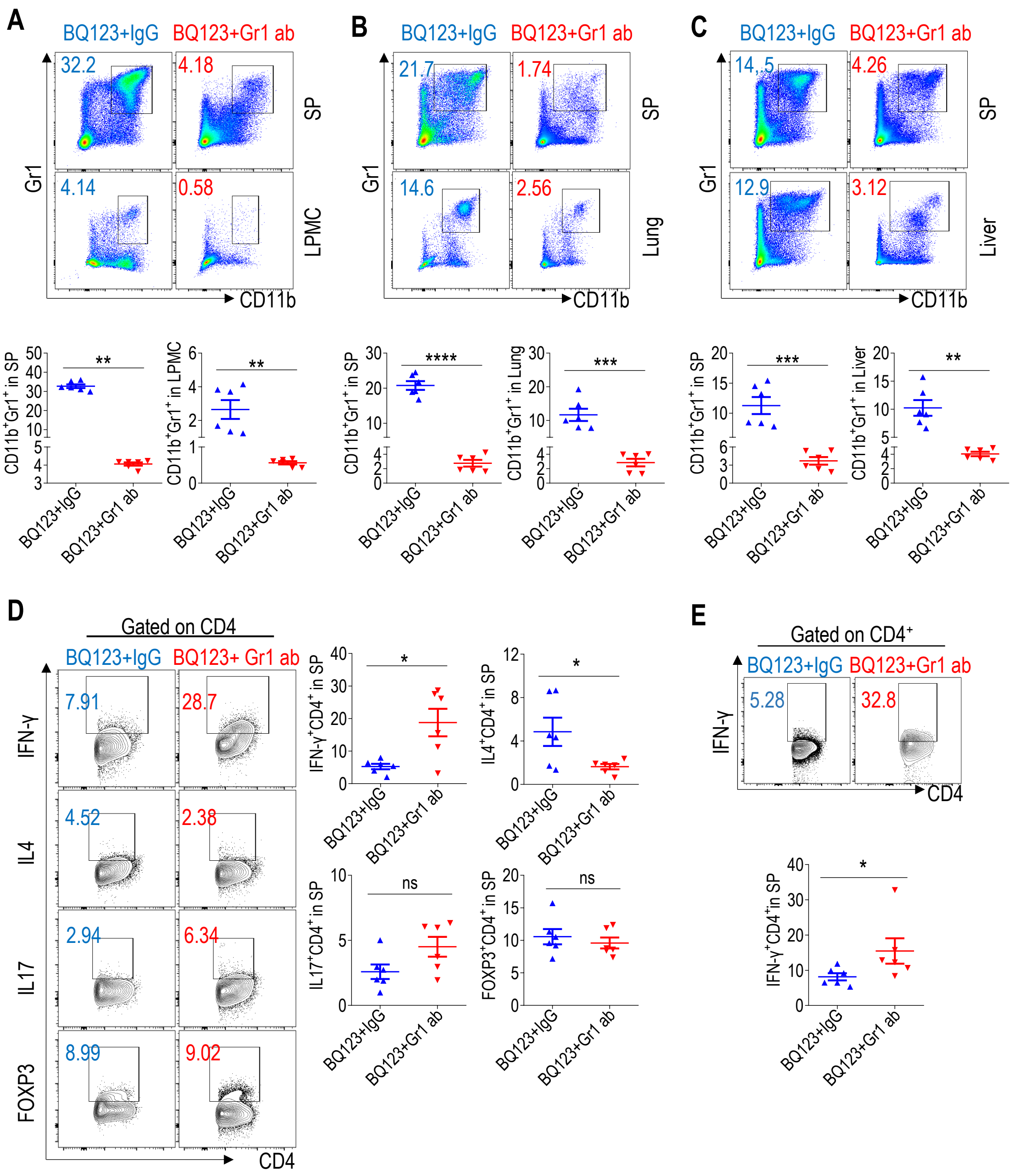
A
BQ123+IgG
BQ123+Gr1 ab
32.2
4.18
SP
4.14
0.58
LPMC
Gr1
CD11b
B
BQ123+IgG
BQ123+Gr1 ab
21.7
1.74
SP
14.6
2.56
Lung
C
BQ123+IgG
BQ123+Gr1 ab
14.5
4.26
SP
12.9
3.12
Liver
CD11b+Gr1+ in SP
CD11b+Gr1+ in LPMC
CD11b+Gr1+ in Lung
CD11b+Gr1+ in Liver
**
***
****
D
Gated on CD4
BQ123+ Gr1 ab
IFN-γ
IL4
IL17
FOXP3
CD4
7.91
28.7
4.52
2.38
2.94
6.34
8.99
9.02
IFN-γ+CD4+ in SP
IL4+CD4+ in SP
IL17+CD4+ in SP
FOXP3+CD4+ in SP
*
ns
E
Gated on CD4+
5.28
32.8
IFN-γ+CD4+in SP

Supplementary Figure 8. The level of MDSC and inflammatory T cells under Gr1ab injection

Mice were divided into two groups and injected with anti-IgG control or anti-Gr1 antibody intraperitoneally. BQ123 or PBS was injected as shown in Figure 6. The proportion of MDSC ($CD11b^{+}Gr1^{+}$) cell populations was tested in the spleen and diseased tissue. (A) The level of MDSC in the spleen and LPMC for DSS-induced acute colitis. (B) The level of MDSC in the spleen and lung for papain-induced acute pneumonia. (C) The level of MDSC in the spleen and liver for ConA-induced acute hepatitis. (D) The level of T cell subsets in spleen in acute colitis mice. (E) The level of Th1 cells in spleen in hepatitis mice. Representative flow cytometry results and statistical analysis are shown (n=6). Data represent the mean ± SEM; *P < 0.05; **P < 0.01; ***P < 0.001, ****P < 0.0005 and ns= not significant, and are based on the Mann-Whitney test (A, B, and E) or the two-tailed unpaired Student's t test (C, and D).

Sup Figure 9

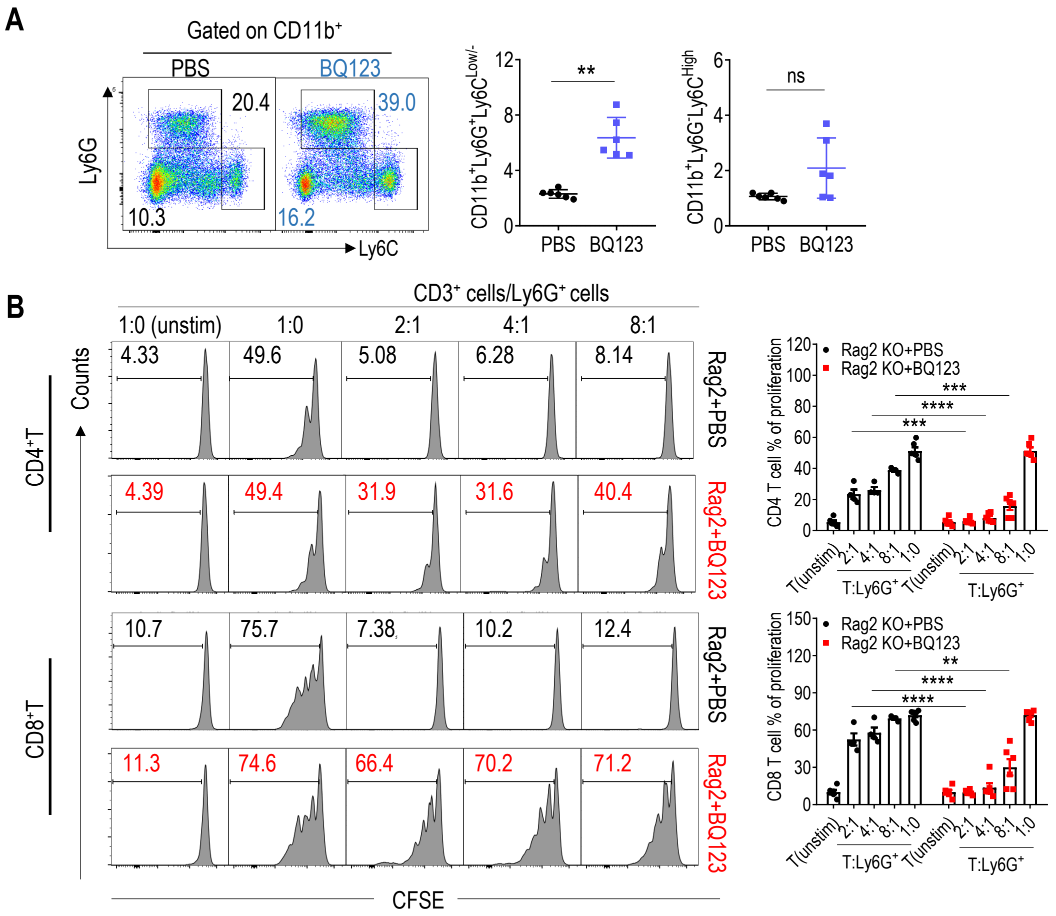

Supplementary Figure 9. BQ123 promoted PMN-MDSC expansion and activation in Rag2 KO Mice

(A) Rag2 KO mice (four-weeks old) were injected with BQ123. PBS was used as a control. All Mice were euthanized on day 9. The proportions of MDSC subsets in the spleen was measured by flow cytometry. A typical example of flow cytometry (left) and statistical results (right) from multiple experiments are shown (n=6). (B) The suppressive activity of BQ123 induced CD11b+Ly6G+Ly6CLow/- cells were determined. CD11b$^+$Ly6G$^+$Ly6C$^{Low/-}$ cells was sorted from the spleen of Rag2 KO mice treated with PBS or BQ123 and co-cultured with CD3+ T-cells isolated from the spleens of BALB/c mice labeled with CFSE at 37°C for 15 min. Cells were plated in a 96-well plate in RMPI-1640 with 10% FBS at different ratios (T/MDSC 1:0, 2:1, 4:1 and 8:1) and stimulated with ConA (5 μg/mL); unstimulated Tcells were used as a negative control. T-cell proliferation was evaluated using CFSE staining. Representative flow cytometry data (left) and statistical results (right) are shown (Rag2 KO +PBS group, n=4; Rag2 KO+BQ123 group, n=6). Data represent the mean ± SEM; **P < 0.01; ***P < 0.001, ****P < 0.0005, and ns= not significant, based on the Mann-Whitney test (A) or the two-tailed unpaired Student’s t test (B)
